# Impact of Future Climate on Radial Growth of Four Major Boreal Tree Species in the Eastern Canadian Boreal Forest

**DOI:** 10.1371/journal.pone.0056758

**Published:** 2013-02-28

**Authors:** Jian-Guo Huang, Yves Bergeron, Frank Berninger, Lihong Zhai, Jacques C. Tardif, Bernhard Denneler

**Affiliations:** 1 Chaire industrielle CRSNG-UQAT-UQAM en Aménagement Forestier Durable, Université du Québec en Abitibi-Témiscamingue, Rouyn-Noranda, Québec, Canada; 2 Département des sciences biologiques, Université du Québec à Montréal, Montréal, Québec, Canada; 3 Centre for Forest Interdisciplinary Research (C-FIR), University of Winnipeg, Winnipeg, Manitoba, Canada; DOE Pacific Northwest National Laboratory, United States of America

## Abstract

Immediate phenotypic variation and the lagged effect of evolutionary adaptation to climate change appear to be two key processes in tree responses to climate warming. This study examines these components in two types of growth models for predicting the 2010–2099 diameter growth change of four major boreal species *Betula papyrifera*, *Pinus banksiana*, *Picea mariana*, and *Populus tremuloides* along a broad latitudinal gradient in eastern Canada under future climate projections. Climate-growth response models for 34 stands over nine latitudes were calibrated and cross-validated. An adaptive response model (A-model), in which the climate-growth relationship varies over time, and a fixed response model (F-model), in which the relationship is constant over time, were constructed to predict future growth. For the former, we examined how future growth of stands in northern latitudes could be forecasted using growth-climate equations derived from stands currently growing in southern latitudes assuming that current climate in southern locations provide an analogue for future conditions in the north. For the latter, we tested if future growth of stands would be maximally predicted using the growth-climate equation obtained from the given local stand assuming a lagged response to climate due to genetic constraints. Both models predicted a large growth increase in northern stands due to more benign temperatures, whereas there was a minimal growth change in southern stands due to potentially warm-temperature induced drought-stress. The A-model demonstrates a changing environment whereas the F-model highlights a constant growth response to future warming. As time elapses we can predict a gradual transition between a response to climate associated with the current conditions (F-model) to a more adapted response to future climate (A-model). Our modeling approach provides a template to predict tree growth response to climate warming at mid-high latitudes of the Northern Hemisphere.

## Introduction

Modeling future growth of forests under climate change is a challenge since our understanding of tree physiology and growth, and the role and rate of genetic adaptation to a rapidly shifting climate is currently limited. Previous modeling studies have attempted to predict potential changes in tree growth, net primary productivity, and forest productivity under increased greenhouse gases emissions scenarios (generally 2×CO_2_) using either empirical/statistical models [1], [2] or process-based models [3], [4]. Tree ring based studies aimed at predicting the impact of future climate usually assume that the relationships between tree growth and climate are linear and constant through time. For example, Chhin et al. [5] using a site-specific empirical regression model, reported negative impact of future climate warming on productivity of lodgepole pine (*Pinus contorta* Dougl. ex Loud. var. *latifolia* Engelm.) in Alberta. Tree ring based studies, however, often do not address the fact that the relationships between growth and climate may change and they often extrapolate growth responses beyond the range where they have been validated.

In this study, we predict future radial growth of trees based on two theoretical assumptions. Given that tree growth conditions may be changing with a warming climate over time, the first assumption is that future response of trees to climate warming at the local scale can be best approximated by the response of trees to climate currently occurring at more southern latitudes. Here we defined this assumption based-model as “Adaptive response model (A-model)”. By shifting the growth model to more southern models as climate warming occurs, the potential effects (e.g., drought effect highlighted in the southern models [6]) will be involved. For example, different climate effects that the northern models may not indicate will be involved, as e.g. trees develop shoots which may be better adapted physiologically for the warming climate. Using the correlation pattern along the gradient 48–50°N in eastern Canada, among sites, between species [black spruce (*Picea mariana* (Mill.) BSP), and jack pine (*Pinus banksiana* Lamb.)], and through time (1825–1993), Hofgaard et al. [7] found significant linear relationship between climate change over time and climate change over latitude. This study supports our first assumption.

In contrast, the second assumption states that future tree growth can be best predicted by the climate-growth equations obtained from the local stands when considering genetic constraints or the lagged effect of genetics on growth. Here we defined this assumption based-model as “Fixed response model (F-model)”. This assumption is supported by Savva et al. [8] who reported in a provenance study that northern populations of jack pine transferred to a southern latitude did not benefit from the warmer climate conditions due to an inherent earlier onset of growth cessation than the local populations. This might indicate a genetic constraint or a lagged effect in genetic response to climate [9] since the onset of bud set and growth cessation, and maximum radial growth rate are genetically-constrained photoperiodic responses [10]. Growth simulations contrasting these two assumptions would provide alternative scenarios for better understanding how future climate warming will impact the growth of trees and forests in eastern Canada.

To accomplish this, we designed a dendrochronological study across a broad latitudinal gradient from 46–54°N in eastern Canada. In our first examination of this data, we systematically investigated the radial growth response of four major boreal species paper birch (*Betula papyrifera* Marsh.), jack pine, black spruce, and trembling aspen (*Populus tremuloides* Michx.) to past climate change in eastern Canada and found their growth responses to the past 50-years of climate warming in this vegetation transition zone differed among species [6]. This broad latitudinal gradient design may provide an analogue of future climate warming given that the northern growth conditions might be changing to the southern ones with warming. In this study, we further forecast the radial growth of these species over this broad spatial scale using the A- and F- models noted above. We hypothesized that 1) the climate-growth calibration models developed from the southern stands can be used to predict future growth of stands growing in the north; 2) the northern trees may be able to benefit from future warming to enhance growth.

The specific objectives of the current paper were fourfold. First, we updated the calibration of the climate-growth models for each species at different latitudes reported in Huang *et al.* [6] through extending the calibration period to the maximum length of chronology and climate data for each species at each site. Second, we forecast the potential mean growth change (MGC) of a species for a given latitude from 2010–2099 based on four different IPCC Emissions Scenarios generated from three General Circulation Models (GCMs) and one from the Canadian Regional Climate Model (CRCM) using the above calibration models. Third, we quantified whether the predicted MGC of a given species at different latitudes differs among different climate change projections using different calibration climate-growth models through decomposition of variance. This process can allow us to determine the most appropriate calibration model for predicting future radial growth at a given latitude over time. Last, we predict the potential MGC of each species for a given latitude from 2010–2099 using the A- and F- models constructed on the two assumptions, respectively. This study expands how we simulate future growth rate of trees and forests to consider both environmental and genetic effects.

## Materials and Methods

### Study Area

**Figure 1:**
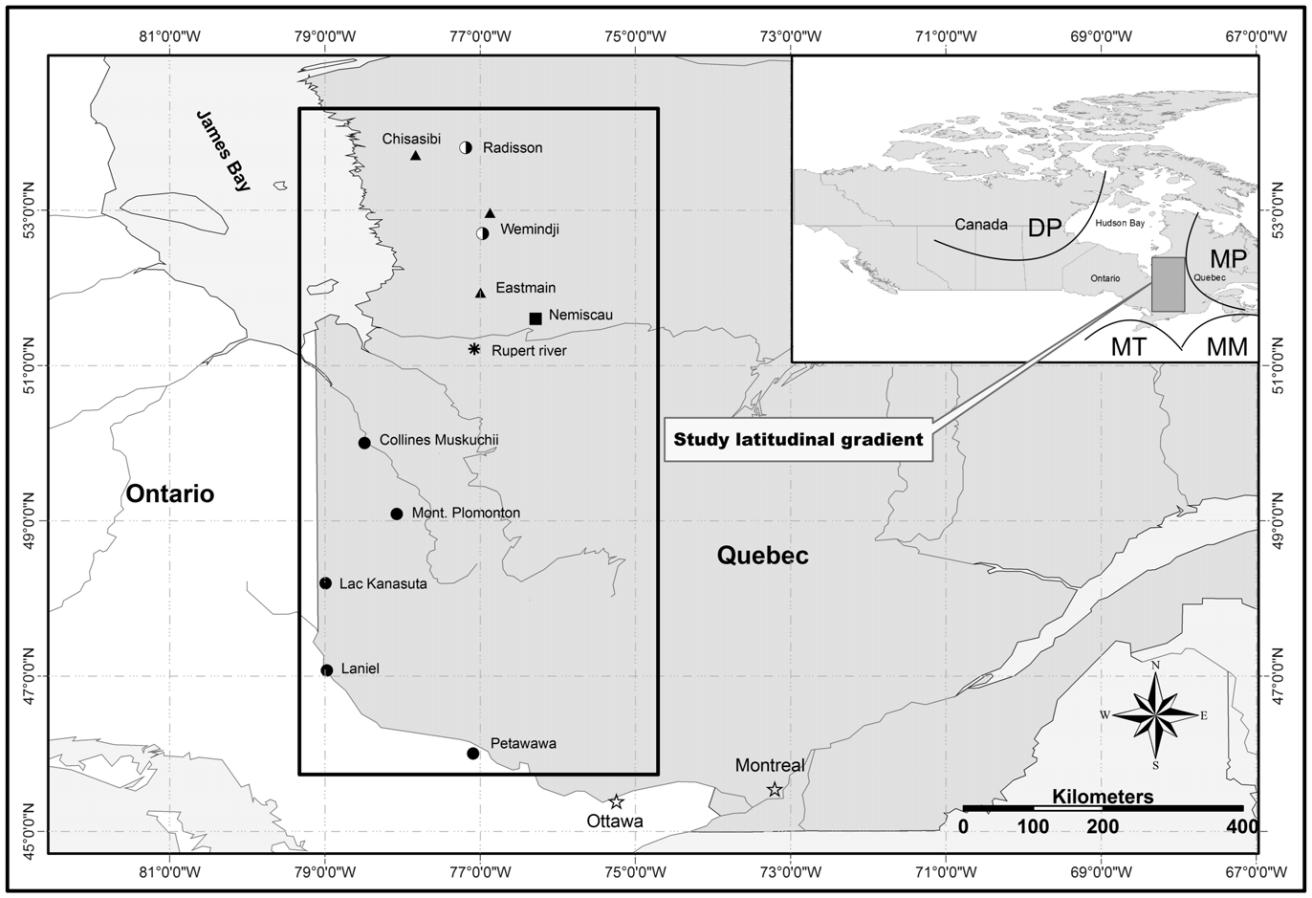
The sampling sites for paper birch, jack pine, black spruce, and trembling aspen in the eastern Canadian boreal forest, where all four species (•), only two conifers (▴), only two deciduous species (half solid circle), only aspen, spruce and pine (*), and only birch (▪) were sampled at the site. The origins of major air mass types affecting the climate of the region are also indicated: dry polar (DP), moist polar (MP), moderate moist (MM), and moist tropical (MT) [11].

The study area is located along the Quebec-Ontario border over a latitudinal gradient ranging from Petawawa (approximately 46°N) in the south to Radisson (approximately 54°N) in the north (Fig. 1). The topography along the gradient is generally flat and uniform with low-elevation hills and rock outcrops. The climate of the region is dominated by dry polar and moderate polar air masses in winter, and by moist maritime and moist tropical air masses in summer [11]. A climate gradient followed the latitudinal gradient, as described in Huang et al. [6]. A vegetation transition zone between the mixedwood and the coniferous-dominated boreal forest occurs at approximately 49°N [12]. The tree line is about 500 km north of the northernmost stands.

### Tree-ring Increment Data

Ring width data sets for aspen, birch, spruce and pine used in this study were exactly the same as reported in Huang et al. [6], i.e. residual chronologies from 8 aspen stands, 8 birch stands, 9 spruce stands and 9 pine stands (2 cores per tree, 20 trees per stand). All tree-ring chronologies were developed according to standard dendrochronological techniques: crossdating, standardization (60-year or more flexible spline detrending was used), calculation of residual ring-width chronologies, as well as reduction of noise created by insect defoliations [6], [13]. All residual chronologies were then used to calibrate the current climate-growth response. The expressed population signal, which is a statistic used to evaluate the reliability of a chronology, was above the generally accepted cutoff value of 0.85 for all time spans of the residual chronologies [14].

### Instrumental Climate Data for Updating Calibration Models

Instrumental climate data used for updating the calibration models at each of 12 locations were interpolated from ANUSPLIN (version 4.3) [15] for the period 1901–2003 (see details in [6]). Climate variables used in the model included monthly maximum temperature (T_max_), monthly minimum temperature (T_min_), and monthly total precipitation (P_total_). In addition, the monthly Canadian Drought Code (CDC) from May to October was also calculated for each climate data set as described by Girardin & Wotton [16] using monthly T_max_ and P_total_ generated by ANUSPLIN. The CDC is a numerical index representing the average moisture content of deep and compact organic layers, and was used to investigate if soil moisture variability had any impact on tree growth in the region.

### Climate Change Projections for Future Growth Simulation

For each of the 12 locations, the simulations obtained from 8 climate change scenarios through three GCMs and the Canadian third-generation coupled global climate model (CRCM3) were used for growth predictions. They included three sets of data generated from CGCM3 under the A1B, A2, and B1 scenarios [17], two sets of data generated from UK Hadley Centre (HadCM3) under the A2 and B2 scenarios [18], two sets of data generated from Max Planck Institut für Meteorologie (ECHAM4) under the A2 and B2 scenarios [19], and one set of data generated from CRCM3 under the A2 scenario. They were referred to CA1B, CA2, CB1, HA2, HB2, EA2, EB2 and MA2 respectively in this paper. A brief description of the four emissions scenarios and the corresponding GCMs were given in Table S1.

The regional projections were used to estimate the climate anomalies using a delta method in which the predicted changes, or anomalies, of climate variables (their mean and variance) obtained from climate future simulations are added to the current climate normal [20]. These anomalies were applied to a series of years randomly selected from 1980 to 2000, as reported by Lapointe-Garant et al. [21]. T_max_ and P_total_ data obtained from this process were then used to calculate the CDC from May to October for each of the 12 locations. All climate change projection data during 1961–2099 were used for future growth simulations.

### Updating Climate-growth Calibration Models using Instrumental Climate Data

Based on tree-ring chronologies and instrumental climate data from 1950–2003, Huang et al. [6] calibrated and reported the climate-growth calibration models for all species/sites over the gradient. In the current study, to obtain the most reliable calibration models for future simulation, it would be preferable to use the maximum length of the chronologies and instrumental climate data to update the calibration models reported in Huang et al. [6]. Least-squares stepwise multiple regression employing a backward selection was used to update these empirical climate-growth models. The covariates included 17 T_max_, 17 T_min_, and 17 P_total_ monthly variables (previous May to current September), and 11 CDC at each latitude/species. This updating procedure resulted in the shortest and longest calibration period being, respectively, 1946–2003 (BS at 46°N) and 1902–2003 (10 latitudes/species, see Table S2). The common longest calibration period among all species/latitudes was 1946–2003.

For each latitude/species, the statistically important monthly climate variables were retained during modeling trials, and multi-month combinations of these climate variables were then calculated to reduce the number of predictors to establish the best calibration model (minimum tolerance P = 0.05). Variance inflation factors (VIF) were also calculated to detect multicollinearity among the variables [22]. VIFs were generally lower than the accepted value of 3. The minimum Akaike Information Criterion (AIC) [23] was used to choose the best calibration model. In general, less than 10 combined climate variables were retained in the final model (Table S3).

The performance of each regression model was cross-validated using a split sample calibration–verification scheme [24]. First, climate data during the full calibration period was split into two subperiods at the year of 1965, thus resulting in a calibration subperiod and a verification subperiod, respectively. For example, when the full calibration period is 1902–2003, then the calibration and verification subperiod is 1902–1965, and 1966–2003, respectively. Second, the regression coefficients obtained from the equation fit to the calibration subperiod were applied to the climate variables over the verification periods to produce a series of tree-ring increment estimates. The same process was repeated, reversing the subperiods for model calibration and model verification. Tree-ring increment estimates obtained from the above procedure were further compared with tree-ring observations to assess the strength of the model using the Pearson R^2^, reduction of error (RE, its values can range from −∞ to a maximum of 1 and any positive value indicates that the model has some skill), product means test (PM), and sign test (ST counts the numbers of agreements/disagreements in sign of deviation from the means in the observed and estimated series) [25]. All analyses were conducted using SAS 10 (SAS Corporation, Cary, North Carolina, USA). The program VFY [26] was used for calculation of the RE, PM and ST verification statistics.

### Mean Growth Change Simulation and Decomposition of Variance

Each of the updated calibration climate-growth models was used to predict ring-width index (dependent variable) for the period 2010–2099 based on each of 8 sets of climate change scenarios data (independent variables). The simulated mean growth change (MGC) was expressed as a percentage of growth (ring-width index) anomalies over 1962–1991. Because we are unclear whether each of the calibration climate-growth models of a species has the same capability to predict future growth, the decomposition of variance [27] was thus performed to quantify whether the predicted MGC of a given species at different latitudes differed among different climate change scenarios using different calibration models. The decomposition of variance was performed on a large dataset of MGC (8 or 9 calibration models ×8 climate change scenarios ×8 or 9 latitudes). The variance of the whole simulated dataset was broken down into different variance parts as follows:
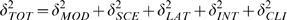



Where *δ^2^*_TOT_ is the variance of the whole simulated growth and *δ^2^*_MOD_ the variance caused by different calibration climate-growth models, *δ* ^2^_SCE_ the variance caused by different climate change scenarios, *δ* ^2^_LAT_ the variance caused by different latitudes at which predictions were made, *δ* ^2^_INT_ the variance caused by interactive effects of the above factors (all possible interactions), and *δ* ^2^_CLI_ the variance that cannot be explained by the above factors, and thus be considered to be caused by future climate variability. The breakdown of variances was calculated using ANOVA.

### Model Construction and Future Mean Growth Change Simulation

#### A-model

Since the first assumption insists that with climate warming the calibration model currently developed for the southern stands could be used to predict future growth of the northern stands, the A-model was constructed as follows. First, a future 30-year mean temperature was calculated for three subperiods 2010–2039, 2040–2069, and 2070–2099, respectively, based on the 8 climate change scenarios and 9 latitudes (Table 1). Second, comparisons between the moving future 30-year mean temperature (e.g., 1981–2010, 1982–2011, and so on) and the past 30-year (1961–1990) ANUSPLIN mean temperature at different latitudes were made to identify the latitude at which future mean temperature at a given latitude for a given subperiod approximates that of a southern stand. Third, the calibration models established from the given northern latitude to the best southern latitude identified above were involved to predict future MGC using 8 sets of climate change scenario data simulated for the given northern latitude, given that those climate conditions might be gradually changing from the given latitude to the best southern latitude with warming over time.

**Table 1:** Comparisons of 30-year mean annual temperatures (±SD) between instrumental climate data for 1961–1990 and future mean GCMs simulation climate data from eight climate change scenarios for 2010–2039, 2040–2069, and 2070–2099 over latitude of 46–54°N, as well as the corresponding models identified and applied at each latitude from 48–54°N.

Latitude (°N)			Time Period		
		1961–1990	2010–2039	2040–2069	2070–2099
46	T (°C)	4.25 (0.65)	5.83 (0.81)	7.31 (0.96)	8.75 (1.14)
	Models	N/A	N/A	N/A	N/A
47	T (°C)	3.02 (0.75)	4.47 (0.84)	5.97 (0.98)	7.45 (1.20)
	Models	N/A	N/A	N/A	N/A
48	T (°C)	1.14 (0.78)	2.66 (0.84)	4.18 (0.97)	*5.67 (1.21)*
	Models		L48-L47	L48-L46	*L47-L46*
49	T (°C)	−0.63 (0.80)	0.82 (0.82)	2.36 (0.96)	3.88 (1.22)
	Models		L49-L48	L48-L47	L48-L46
50	T (°C)	−0.89 (0.85)	0.67 (0.85)	2.24 (0.97)	3.79 (1.25)
	Models		L50-L48	L49-L47	L48-L46
51	T (°C)	−1.54 (0.92)	0.13 (0.85)	1.73 (0.94)	3.32 (1.23)
	Models		L51-L48	L49-L47	L48-L46
52	T (°C)	−3.06 (1.00)	−1.28 (0.89)	0.38 (0.97)	2.02 (1.27)
	Models		L52-L50	L51-L48	L49-L47
53	T (°C)	−3.21 (1.01)	−1.42 (0.90)	0.23 (0.98)	1.89 (1.28)
	Models		L53-L50	L51-L48	L49-L47
54	T (°C)	−3.18 (1.07)	−1.35 (0.94)	0.34 (1.02)	2.03 (1.32)
	Models		L54-L50	L51-L48	L49-L47

*Note:* Models at 46°N and 47°N were not determined (N/A); The underscored italic text indicates the simulations with uncertainties. Standard deviation (SD) of climate projections is interannual variation of the average of the mean of the 8 climate change scenarios.

The calibration models identified in above procedure for each latitude/species are listed in Table 1. In many cases several models were involved for future growth simulation. For instance, the growing condition of aspen at 54°N during the future subperiod 2010–2039 is assumed to be gradually changing to that at a more southern latitude (e.g., 50°N) when the 2010–2039 mean temperature at 54°N is near the 1961–1990 mean temperature of 50°N (southern limit). In this case, the calibration models from 54, 53, 52, 51 and 50°N were all selected for the 2010–2039 growth simulations of stands at 54°N. We calculated the growth response by interpolating the growth models based on average yearly temperatures. For the next subperiod (e.g., 2040–2069), the southern limit chosen above (e.g., 50°N) was considered as the northern limit, and a more southerly site yet (than 50°N) was chosen, and the calibrated models established between these two limits were used sequentially for future growth simulation. The same principle was applied to each of all the latitudes/subperiods. However, when future climate range was beyond the range of our data (such as for 46–47°N and during 2070–2099 at 48°N, Table 1), extrapolation was thus not performed and future growth in these latitudes were not further discussed. The 95% confidence interval was built for each prediction, which indicates the uncertainty due to different models and climate scenarios. Decomposition of variance was also calculated.

#### F-model

Our second assumption was that future tree growth would be best predicted by the climate-growth equations obtained from the given local stands due to their local genetic constraints. The F-model was constructed and future growth was simulated. This was a simpler process where the calibrated climate-growth model obtained for each species in each local stand was employed to simulate future MGC based on each of 8 climate change scenario data simulated for the given latitude. The MGCs calculated for the 8 climate change scenarios were further averaged for comparison with the A-model simulations. The 95% confidence interval was also built, which indicates the uncertainty due to climate scenarios.

## Results

### Updated Climate-growth Calibration Models

As listed in Table S2, the significant and positive RE values reveal that the models are reasonably robust over the full length of the calibration period. Both the PM and ST results suggest significant predictive skills to reproduce the magnitude and direction of year-to-year changes. Among four species, higher R^2^ and RE values in most of pine stands along the gradient indicate that pine models are more robust than that of the other species, and should have high fidelity to predict radial growth. As shown in Table S3, the main climate variables selected for aspen models mostly include previous summer- and autumn temperatures, current growing season temperatures and current precipitation. The main climate variables for birch models include previous autumn temperatures and current spring and summer temperatures. The models for two conifers highlight mostly winter and the average growing season temperatures.

### Future Climate Anomalies

As shown in Fig. S1 and Table S4, compared to mean climate during 1961–1990, the greatest positive temperature anomalies (+2 to +6°C) were predicted by ECHAM4 (EA2 and EB2), and the smallest positive anomalies (+1 to +4°C) were simulated by HadCM3 (HA2 and HB2). Higher precipitation anomalies (+40 to +180 mm) were predicted by CGCM (CA1B, CA2, and CB1) and CRCM3 (MA2) than that predicted by ECHAM4 and HadCM3. Severe drought anomalies (+10 to +40 units) were more frequently predicted by ECHAM4 (EA2 and EB2) than other models.

### Mean Growth Change and Decomposition of Variance

**Figure 2:**
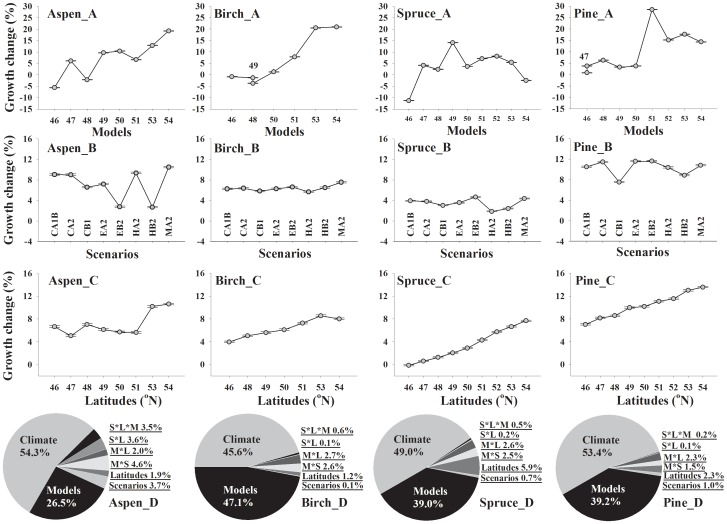
Partitioning of variance in mean simulated growth change of paper birch, jack pine, black spruce, and trembling aspen along the gradient. The pie plot shows that the proportions of variance in mean growth change for each species explained by the calibration models (M), scenarios (S), latitudes (L), and the interactive effects of the above three factors, as well as the climate. Grey dots represent the least square means of potential growth change of each species predicted from 2010 to 2099. Grey short lines represent the standard error of the means.

As illustrated in Fig. 2 D, climate variability accounts for about half of the simulated variability (45.6%–54.3%), followed by the calibration model differences representing 26.5%–47.1% of simulated variability, scenario differences representing 0.1%–3.7%, latitude differences representing 1.2%–5.9%, and other interactive effects representing 4.2%–13.7%. As shown in Fig. 2 A, the northern models for aspen, birch and pine generally predicted a large growth increase, whereas the southern models predicted a minor growth change. The models for spruce mostly show a moderate increase in growth with increasing latitude except for the southernmost and northernmost stands. Among different latitudes (Fig. 2 C), the MGC was predicted to increase towards the north (5.1% to 10.6% from south to north for aspen, 4.0% to 8.6% for birch, −0.2% to 7.7% for spruce, and 7.0% to 13.6% for pine). Among different scenarios (Fig. 2 B), relatively less variability in simulated MGC was found. Within the GCMs, the A2 scenario generally resulted in better growth in aspen and pine than the B2 scenario, yet no such difference was found for other two species.

### Mean Simulated Growth Change using the A-model

**Figure 3:**
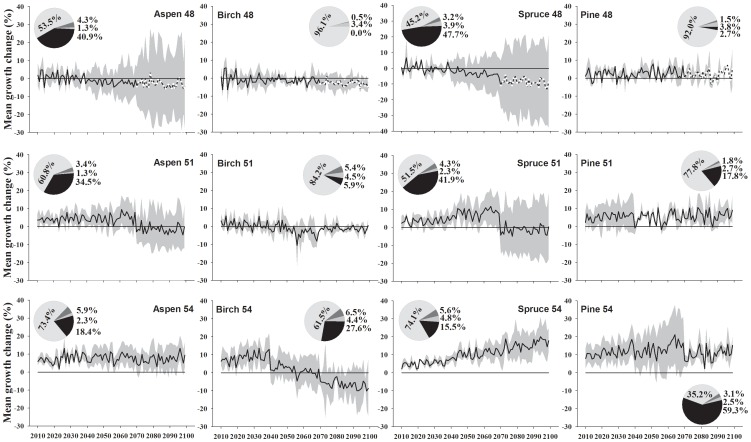
The predicted mean growth change of trembling aspen, paper birch, black spruce, and jack pine at 48, 51 and 54°N under the A-model. The pie plots indicate the proportion of variance in mean potential growth change explained by, counter clock wise, the calibration models (dark), scenarios (grey), calibration models *scenarios (darker grey), and climate (blue). The grey zones are 95% confidence interval. White dashed lines indicate the estimation of mean growth change with uncertainties.

**Figure 4:**
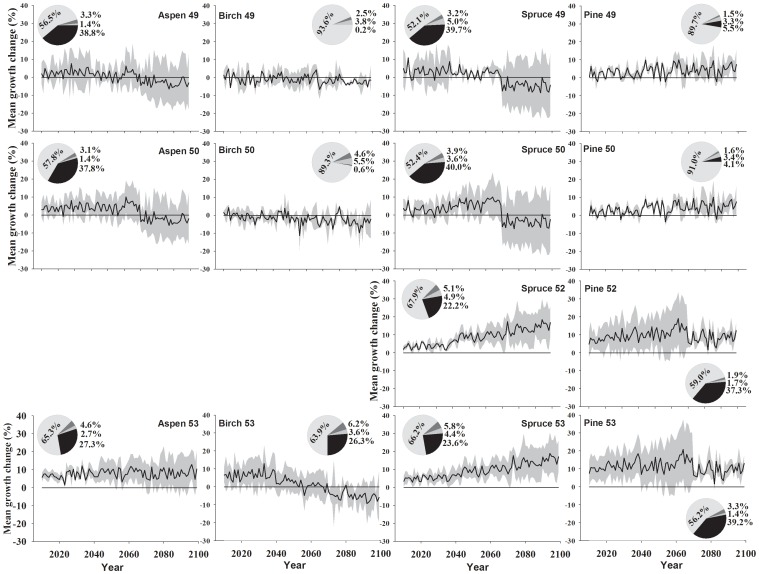
The predicted mean growth change of trembling aspen, paper birch, black spruce, and jack pine at 49, 50, 52 and 53°N under the A-model. The pie plots indicate the proportion of variance in mean potential growth change explained by, counter clock wise, the calibration models (dark), scenarios (grey), calibration models *scenarios (darker grey), and climate (blue). The grey zones are 95% confidence interval. White dashed lines indicate the estimation of mean growth change with uncertainties.

As shown in Fig. 3 and Fig. 4, the A-model simulation results indicate that aspen would have a positive MGC (10–15% growth increase) during 2010–2099 at 53–54°N, and a moderate growth increase (less than 10%) during 2010–2069 followed by a growth decrease (less than 10%) during 2070–2099 at 50–51°N. For stands at 49°N, a moderate growth increase (less than 8%) during 2010–2049 was predicted, followed by growth fluctuations (increases/decreases) during 2050–2069 and a growth decrease (less than 10%) after 2070. For stands at 48°N, a low growth increase (less than 5%) during the next one or two decades followed by a gradual growth decrease thereafter were simulated, with a large 95% confidence interval. As shown by the pie plot in Fig. 3 and Fig. 4, the results of partitioning the variance showed that from south to north the variance in simulated MGC explained by climate variability increases (53.5% to 73.4%), whereas that explained by the calibration models decreases (40.9% to 18.4%). Both together accounted for the largest proportion of the variance, yet other factors like climate change scenarios and scenario×calibration model interactions explained only very little variance.

Birch would show moderate growth increases (less than 20%) until 2040s followed by a gradual growth decrease (less than 20%) thereafter at 53–54°N. For stands at 51°N, a weak growth increase (less than 10%) during 2010–2039 was predicted, followed by gradual growth decrease thereafter. Stands at 48–50°N would show a moderate growth decrease (less than 10%) during 2010–2099. Partitioning of variance indicates that the climate variability explained more variance for stands at 48–51°N (84.2% to 96.1%) than that for stands at 53–54°N (61.5% to 63.9%), whereas the calibration models explained less variance for stands at 48–51°N (0.0% to 5.9%) than that for stands at 53–54°N (26.3% to 27.6%). Other factors explained only very little variance.

Spruce at 52–54°N was predicted to show an obvious growth increase (up to about 20%) until 2099. For stands at 49–51°N, a growth increase (less than 15%) during 2010–2069 was simulated, followed by a moderate growth decrease during 2070–2099, with a large 95% confidence interval. Stands at 48°N would show a weak growth increase (less than 6%) during the coming decade, followed by a gradual growth decrease thereafter. The results of partitioning of variance showed that the variance in simulated MGC explained by climate variability increases from 45.2% to 74.1%, and that explained by the calibration models decreases from 47.7% to 15.5%, from south to north, respectively. Other factors explained only very little variance.

In contrast to the previous three species, pine at all latitudes would show a consistent growth increase until 2099. Up to 20% growth increase in stands at 52–54°N and up to 10% growth increase in stands at 48–51°N are expected. The results of partitioning of variance showed that, from south to north, the variance in simulated MGC explained by the climate variability decreases from 92.0% to 35.2%, and explained by the calibration models increases from 2.7% to 59.3%. Other factors also explained very little variance.

### Mean Simulated Growth Change using the F-model

**Figure 5:**
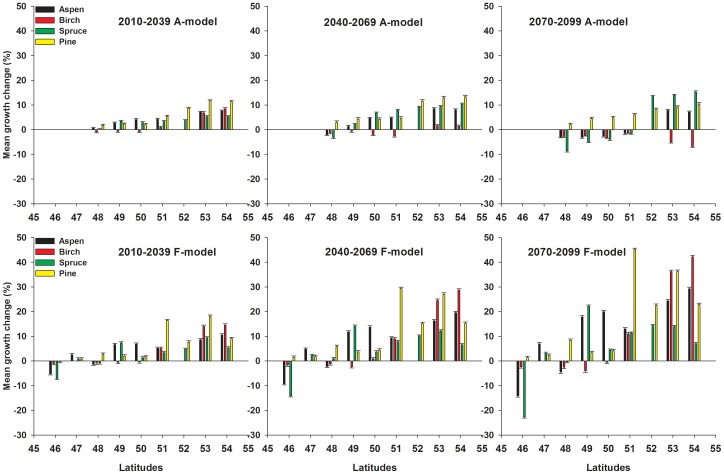
Comparison between average growth change of paper birch, jack pine, black spruce, and trembling aspen predicted by the A-model, and that predicted by the F-model at each latitude from 48 to 54°N over time slices 2010–2039, 2040–2069, and 2070–2099 in the eastern Canadian boreal forest. The error bars were shown by the short dashed lines.

As illustrated in Fig. 5 and Fig. S2, the F-model simulation results showed that except for an expected growth decrease until 2099 at 46 and 48°N, aspen would have a moderate growth increase during 2010–2099 at most of the latitudes, with the highest growth increase (up to 40%) at 53–54°N. Birch would show a potential growth increase until 2099 at 51–54°N, and a growth decrease during most of the 21^st^ century at 46–50°N. Like aspen, the fastest growth increase (up to 40–50%) is expected for stands at 53–54°N. Spruce would show a moderate growth increase north of 48°N, relatively minor growth fluctuations at 47–48°N, and a linear growth decrease at 46°N to as low as 30% in 2099. Pine would show a strong linear growth increase (up to 60%) at 51–54°N, moderate growth increases (10%) at 48–50°N, and a weak, fluctuating growth increase from 2010–2099.

## Discussion

Our simulations showed that tree ring based growth simulations depend strongly on the assumptions of the growth model. There were consistent and large differences between the A- and the F- model. Further differences were, however, caused by the climate change scenarios and natural climate variability.

### Decomposition of Variance

The decomposition of variance showed that climate variability will remain a major source of growth variation. There are large differences between the various growth models, indicating that our poor understanding of the growth responses is a second important source of uncertainty. Surprisingly, the choice of the climate scenario and the latitude was not such an important effect. We should also note that the decomposition of variance deals with variations of simulated growth indices. These exclude possible interactions of the environment with site factors such as soils. Furthermore, other factors such as insect outbreaks might change the growth [28].

### Comparison between the A-model and F-model

Species adjustments to environmental change occur in the short term through physiological plasticity of individuals and in the long term through the evolutionary process of selection, migration, mutation, and drift [29]. With rapid warming predicted in the current century, slow maturing organisms like trees may not be able to keep pace with this change [30]. Evolution and migration may, therefore, play a minor role in the survival of tree species, whereas phenotypic variation may be the most important key process [9]. The main feature of the A-model is that it varies over time. That is, a gradual changing environment for tree growth is highlighted because more southern models involved may highlight different growth responses to climate over spatial gradient. This modeling concept, i.e., a changing environment, is similar to the provenance experiments that often transfer a northern original population (seeds or seedlings) to a more southern location to detect its fitness compared to the local populations [31]. Compared to the provenance experiments, however, our modeling approach has the advantages of sampling at a broad spatial scale and short duration, focusing on mature trees and forests, with much less cost. Its disadvantage is that it cannot separate environmental from genetic effects like a provenance trail does. Consequently, this model might reveal an alternative scenario of growth when tree growth conditions will be changing with warming.

In contrast, the F-model is similar to the previous empirical tree-ring modeling studies that often used the local models to simulate future tree growth under climate change scenarios [5], [32]. Although this approach is identical to methods that use static and linear relations of climate and tree growth to extrapolate future growth trends, its innovative aspect is the ecological interpretation of the approach, not the approach itself. Common garden experiments showed that the null transfer for most of forest tree species studied in North America is optimal for current climates in terms of temperature, suggesting that local populations generally have higher fitness than non-local populations in local climates [33], [34]. Therefore, the local model established from the current stands might be the best model to quantify the growth response to a rapidly changing climate. Growth responses of trees in the model appear to be generally larger and the model seems to emphasize strong temperature responses of northern populations and to put little weight on the effects of drought on tree growth.

A common feature of these two models is that their northern forests all experience a large growth increase, but minor growth changes were predicted for southern forests. This is in agreement with the principle of the limiting factors that has been commonly used in dendrochronology [35]: with increasing latitude, temperature plays a more important role in limiting radial growth, whereas synergistic factors (temperature, precipitation, drought) are more significant in the south [36]. Hence, with climate warming, the northern stands would lose their temperature limitations, leading to a large growth increase due to extension of the growing season, earlier budbreak and growth stimulation, as well as less damage by severe cold temperatures; The southern stands would become more drought stressed, thus tending to show minor growth change [36]. Generally speaking, in the north where tree populations are occupying climates much colder than their optima, trees should have the smallest losses but more gain in growth with climate warming, whereas in the south, where the discrepancies between the inhabited and optimal environments are the least, the negative effect of a warming climate will be the greatest [33]. Rehfeldt et al. [33], for instance, predicted more gains in productivity of the *P. contorta* Doug. ex Loud. forests of British Columbia at high latitudes and elevations, which would overcompensate the losses projected for lower elevations in the south under future warming.

### Simulation Output from the A-model and F-model

Future consistent growth increases in northern stands predicted by the two models are in agreement with the findings of provenance experiments [9], [37] and forest growth modeling studies [21], [38]. For instance, Rehfeldt *et al*. [9] reported that the immediate short-term response of *P. sylvestris* to global warming should be strongly positive for populations inhabiting severe climates. Thomson et al. [37] documented that the northern black spruce provenances in Ontario currently achieve better height growth when moved to more southern locations. Eggers et al. [38] projected the forest resources from 2000 to 2100 for 15 European countries under different climate scenarios, and observed significantly increased growth in northern Europe, but minor growth change in southern Europe.

Consistent growth decreases or minor growth change forecasted in southern stands by the two models are supported by the current provenance experiments and other modeling studies [9], [39]. For example, several provenance experiments on black spruce and jack pine in Ontario revealed that their current maximum growth is achieved in the central latitudes (approximately between 45 and 48°N) [37], [40]. Potential drought or heat stress -induced growth decline will be expected with future warming [41]. Rehfeldt et al. [9] pointed out that climate warming would negatively influence the southern populations of *P. sylvestris* that currently inhabit mild climates. Our results are also supported by other studies in Europe [42] and North America [36], [43] documenting drought-caused growth decline or loss in several boreal tree species in southern part of their range. Compared to the other species, consistent growth increase predicted for the southern pine stands during 2010–2099 might be due to its better drought-tolerance than others or more variance explained in the model.

### Rationale of the Predicted Future MGC Under the Two Models

Under the A-model, all stands from 48 to 54°N were predicted to show moderate growth increase, then a decrease with time rather than a steep linear growth change over time. These results corroborate tree physiological studies which show that the optimum growth of trees often occurs in an intermediate climate conditions (moderately warm and moist) [44]. Therefore trees might not be able to continue to enhance growth after reaching the optimum during the latter part of the current century. Furthermore, future climate-induced changes in disturbance regimes such as insect outbreaks might also have a negative effect on growth [45]. Last, but not least, this moderate growth change calculated through means of growth estimates obtained by several linear models from different latitudes is equivalent to that estimated on the same aspen dataset by a nonlinear model, which integrated both climate and non-climate factors [21]. Overall the A-model might indicate a lower boundary for future growth.

In contrast, simulated growth change under the F-model showed high variability and large range of growth responses across species/stands. It assumes that actually, the current populations at mid to high latitudes established after a long evolutionary process and thus are close to their optimal growth conditions [33]. This assumption has also been supported by the provenance trial studies that observed growth decrease when moving the populations growing at mid latitude to more southern latitudes [8], [37], [40]. The conditions for the near optimal growth would persist only in the next decades; thereafter tree growth would decrease with climate warming. The climate-growth response model established at the local stands would thus be almost the best model and may give best growth prediction during the current century. In any case, the simulated large growth change under the F-model is based on the assumption that future climate-growth relationships would be constant over time. In fact, this would be possible only during the coming decades and the models will be very likely to shift from the northern ones to more southern ones with warming, i.e., growth simulation under the A-model. Hence the F-model prediction might indicate an upper boundary for future growth. Altogether, we infer that under climate warming, potential MGC of these four species in eastern Canada might be somewhere between the two simulation results obtained by the A-model and F-model, respectively.

### Mechanism of Adaptation, Acclimation and Plasticity

Aspen, birch, spruce, and pine are typically fire-adapted, wind-pollinated species, and bear high genetic variability [34], [46]. The trees currently growing at the sites have genetically adapted to the local environmental and climate conditions, and would not be able to fully adapt to future climatic conditions considering the rate of changes and the pace of genetic selection [34], [47]. However, they can demonstrate some growth plasticity to respond to climate warming such as early onset of xylem cell production [48], [49], spring early flowering, budburst, and shoot extension [49], [50]. As the next generation develops acclimation of seeds to new climatic environment and selection of best adapted genotypes will contribute to a better response to the changing climate. Common garden experiments have shown high among-population levels of genetic variation for quantitative traits associated with adaptation, geographic climatic gradients, and genotype-by-environment interactions [34], providing strong evidence of local adaptation of populations to climate [51]. Therefore, as time elapses we can predict a gradual transition between a response to climate associated with the current conditions (the F-model) to a more adapted response to the future climate (the A-model). It may take several generations and a constant climate for trees to reach an optimal response to future climate.

### Model Limitations

Our simulation results might be biased by different periods of calibration for different tree species/latitudes or lower explained variance in the calibration model (adjR^2^). Future predictions might be also influenced by other external factors that were not involved in the models, such as fire disturbances [16,], species competition [52], as well as the potential for a direct CO_2_ fertilization effect [53] or possible nitrogen deposition [54]. Moreover, choosing a calibration model derived from the southern populations for predicting a northern population neglects the genetic differences (in contrast to the phenotypic differences). However, if the southernmost populations are already genetically adapted but the northernmost populations are not able to adapt to the warming by 2099, there would be genetic differences between the southernmost and northernmost populations. As a result, the southernmost models-based growth prediction for the northern population would have already included genetic differences in the simulation. Therefore the current approach does not conclusively allow for disentangling genotypic and phenotypic variation. Finally, future growth change prediction was based on the standardized tree-ring indices, not the absolute growth change in ring width. Actual growth can be calculated through multiplying mean annual ring-width growth during 1961–1990 with the percentage change predicted by the two models (Table S5).

### Implications

Overall, the main trend demonstrated by both the A- and F-model simulation results is that stands to the north of the vegetation transition zone of 49°N would mostly increase growth, whereas those at south of 49°N would decrease growth in the current century. In terms of species, the results indicate that pine and birch might benefit the most and least, respectively, from climate warming, and spruce and aspen are intermediate species. Our results suggest that climate warming will not favour deciduous species in eastern Canada in the long term, though it may for the next few decades. This result is contrary to the general expectation of increased growth of boreal deciduous species [55]. Our results altogether further suggest that there might be a potential shift in forest composition and structure from south to north. That is, the southern boreal mixedwood forest might gradually develop into the coniferous-dominated boreal forest with warming over time. As a consequence, forest productivity might be increasing in the north but decreasing in the south. This will further have profound impacts on the sustainable forest management and boreal carbon cycles and equilibrium. Our modeling approach and concept in the study could be used as a template to investigate growth response of other tree species to climate warming at mid-high latitudes of the Northern Hemisphere, which may allow us to further quantify how forest growth, structure, and composition will respond to and shift in a future warming climate.

## Supporting Information

Figure S1 **Positive climate anomalies of future three subperiods 2010–2039, 2040–2069, and 2070–2099 (from bottom to top) from each model in relative to mean climate during the period 1961–1990 (See Table S4 for the reference values) along the latitudinal gradient in eastern Canada.** Abbreviations: Tmax (anomalies for mean maximum temperature during each subperiod), Tmin (anomalies for mean minimum temperature during each subperiod), Precipitation (anomalies for mean total precipitation during each subperiod), drought code (anomalies for mean drought code during each subperiod). See definition for scenarios abbreviations in the text. The climate anomalies values were shown in the figures.
(TIF)

Figure S2 **The predicted mean growth change of trembling aspen, paper birch, black spruce, and jack pine at 46–54°N under the F-model.** The calibrated model for paper birch at 47°N was not established and thus the predicted mean growth change was not shown.
(DOCX)

Table S1 **General circulation models (GCM) including Canadian third-generation coupled global climate model (CGCM3), UK Hadley Centre HadCM3, Max Planck Institut für Meteorologie ECHAM4 and Canadian Regional Climate Model (CRCM3), their corresponding scenarios and the storylines (from the worst to the best) that describe the relationships between the forces driving greenhouse gas and aerosol emissions and their evolution during the 21st century applied in this study.**
(DOCX)

Table S2 **Statistics of the model calibration and verification for trembling aspen, paper birch, black spruce and jack pine along the gradient.** Significance level is at p<0.5. *Note:* r: correlation coefficient; R^2^: explained variance; adjR^2^: square of the multiple correlation coefficients following adjustment for loss of degrees of freedom; SE: standard error of the predictions; RE: reduction of error statistic, which is a measure of shared variance between the actual and modelled series, but is usually lower than the calibration R^2^. A positive value signifies that the regression model has some skill [24]. PM: product means test [35]; ST: sign test [35]. The italic texts indicate insignificant values.
(DOCX)

Table S3 **The calibrated full-period climate-growth (Tree-Ring Index, TRI) models for trembling aspen, paper birch, black spruce, and jack pine along the latitudinal gradient from 46°N to 54°N.** *Note*: The chronology full period and adj R^2^ of each model were listed in Table S2. Monthly climate variables were abbreviations in the model, for example climate variables in May, p5p and p5 indicates precipitation in the previous and current May, respectively; tmax5p and tmax5 indicates maximum temperature in the previous and current May, respectively; tmin5p and tmin5 indicates minimum temperature in the previous and current May, respectively; dc5p and dc5 indicates drought code in the previous and current May.
(DOCX)

Table S4 **The mean climate values (±SD) during the reference period of 1961–1990 over the latitudinal gradient 46–54°N in eastern Canada.** *Note*: Max T and Min T: Mean maximum, and minimum temperature during 1961–90; Precipitation: mean total annual precipitation during 1961–90; Drought code is mean drought code during 1961–90.
(DOCX)

Table S5 **Mean annual ring width [RW (±SD) mm] growth of the four species from 1961 to 1990 over the latitudinal gradient 46–54°N in eastern Canada.** *Note:* NA indicates that stands were not found at 52°N.
(DOCX)

## References

[1] Rathgeber C, Nicault A, Guiot J, Keller T, Guibal F, et al (2000) Simulated response of *Pinus halepensis* forest productivity to climatic change and CO_2_ increase using a statistical model. Global Planet Change 26: 405–421.

[2] Girardin MP, Raulier F, Bernier PY, Tardif JC (2008) Response of tree growth to a changing climate in boreal central Canada: A comparison of empirical, process-based, and hybrid modelling approaches. Ecol Model 213: 209–228.

[3] Running SW, Coughlan JC (1988) A general model of forest ecosystem processes for regional applications I. Hydrologic balance, canopy gas exchange and primary production processes. Ecol Model 42: 125–154.

[4] Berninger F, Nikinmaa E (1997) Implications of varying pipe model relationships on Scots Pine growth in different climates. Funct Ecol 11: 146–156.

[5] Chhin S, Hogg EH, Lieffers VJ, Huang SM (2008) Potential effects of climate change on the growth of lodgepole pine across diameter size classes and ecological regions. For Ecol Manag 256: 1692–1703.

[6] Huang JG, Tardif J, Bergeron Y, Denneler B, Berninger F, et al (2010) Radial growth response of four dominant boreal tree species to climate along a latitudinal gradient in the eastern Canadian boreal forest. Global Change Biol 16: 711–731.

[7] Hofgaard A, Tardif J, Bergeron Y (1999) Dendroclimatic response of *Picea mariana* and *Pinus banksiana* along a latitudinal gradient in the eastern Canadian boreal forest. Can J For Res 29: 1333–1346.

[8] Savva Y, Denneler B, Koubaa A, Tremblay F, Bergeron Y, et al (2007) Seed transfer and climate change effects on radial growth of *P. banksiana* populations in a common garden in Petawawa, Ontario, Canada. For Ecol Manag 242: 636–647.

[9] Rehfeldt G, Tchebakova NM, Parfenova YI, Wykoff WR, Kuzmina NA, et al (2002) Intraspecific responses to climate in *Pinus sylvestris* . Global Change Biol 8: 912–929.

[10] Körner C, Basler M (2010) Phenology under global warming. Science 327: 1461–1462. 20299580 10.1126/science.1186473

[11] Sheridan SC (2002) The redevelopment of a weather-type classification scheme for North America. Int J Climat 22: 51–68.

[12] Gauthier S, De Grandpre L, Bergeron Y (2000) Differences in forest composition in two boreal forest ecoregions of Quebec. J vegetation Sci 11: 781–790.

[13] Huang JG, Tardif J, Denneler B, Bergeron Y, Berninger F (2008) Tree-ring evidence extends the historic northern range limit of severe defoliation by insects in the aspen stands of western Quebec, Canada. Can J For Res 38: 2535–2544.

[14] Wigley TML, Briffa KR, Jones PD (1984) On the average value of correlated time series, with applications in dendroclimatology and hydrometeorology. J Climate Applied Meteorology 23: 201–213.

[15] Hutchinson MF (2004) ANUSPLIN Version 4.3. Center for Resource and Environmental Studies, Australian National University, [WWW document] URL Available: http://fennerschool.anu.edu.au/publications/software/anusplin.php Accessed: 12 September 2011].

[16] Girardin MP, Wotton BM (2009) Summer moisture and wildfire risks across Canada. J Applied Meteorol Climatol 48: 517–533.

[17] Flato GM, Hibler WD, Lee WG (2000) The Canadian center for climate modelling and analysis global coupled model and its climate. Climate Dyn 16: 451–467.

[18] Collins M, Tett SFB, Cooper C (2001) The internal climate variability of HadCM3, a version of the Hadley Centre coupled model without flux adjustments. Climate Dyn 17: 61–81.

[19] Roeckner E, Arpe K, Bengtsson L, Christoph M, Claussen M, et al.. (1996) The atmospheric general circulation model ECHAM-4: model description and simulation of present-day climate. Report No. 218. Max Planck Institute for Meteorology, Hamburg, Germany.

[20] Ramirez-Villegas J, Jarvis A (2010) Downscaling global circulation model outputs: The Delta method decision and policy analysis working paper No.1. Available: WWW document] URL http://www.ccafs-climate.org/downloads/docs/Downscaling-WP-01.pdf Accessed: 2 January 2013.

[21] Lapointe-Garant MP, Huang JG, Gea Izquierdo G, Raulier F, Bernier P, et al (2010) Use of tree rings to study the effect of climate change on trembling aspen in Quebec. Global Change Biol 16: 2039–2051.

[22] Belsley DA, Kuh E, Welsch RE (1980) Regression diagnostics: Identifying influential data and sources of collinearity. John Wiley, New York, USA.

[23] Akaike H (1974) A new look at the statistical model identification. IEEE Transactions on Automatic Control 19: 716–723.

[24] Cook ER, Kairiukstis L (1990) Methods of Dendrochronology: applications in the environmental sciences. Kluwer Academic Publishers, Dordrecht, The Netherlands.

[25] Cook ER, Briffa KR, Jones PD (1994) Spatial regression methods in dendroclimatology: a review and comparison of two techniques. Int J Climat 14: 379–402.

[26] Holmes RL (1999) Dendrochronology Program Library. Laboratory of Tree-Ring Research, University of Arizona, Tucson, Arizona.

[27] Lawler JJ, Edwards Jr TC (2006) A variance-decomposition approach to investigating multiscale habitat associations. Condor 108(1): 47–58.

[28] Drobyshev I, Simard M, Bergeron Y, Hofgaard A (2010) Does soil organic layer thickness affect climate-growth relationships in the black spruce boreal ecosystem? Ecosystems 13(4): 556–574.

[29] Futuyma DJ (1979) Evolutionary biology. Sinauer Associates, Inc, Sunderland, MA, 565p.

[30] Ledig FT, Rehfeldt GE, Sáenz-Romero C, Flores-López C (2010) Projections of suitable habitat for rare species under global warming scenarios. Amer J Botany 97: 970–987. 21622467 10.3732/ajb.0900329

[31] Rehfeldt G, Tchebakova NM, Milyutin LI, Parfenova EI, Wykoff WR, et al (2003) Assessing population response to climate in *Pinus sylvestris* and *Larix* spp. of Eurasia with climate-transfer models. Eurasian J For Res 6: 83–98.

[32] Laroque CP, Smith DJ (2003) Radial-growth forecasts for five high-elevation conifer species on Vancouver Island, British Columbia. For Ecol Manag 183: 313–325.

[33] Rehfeldt G, Ying CC, Spittlehouse DL, Hamilton Jr DA (1999) Genetic responses to climate in *Pinus contorta*: niche breadth, climate change, and reforestation. Ecol Monogr 69: 375–405.

[34] Aitken SN, Yeaman S, Holliday JA, Wang T, Curtis-McLane S (2008) Adaptation, migration or extirpation: climate change outcomes for tree populations. Ecol Appl 1: 95–111. 10.1111/j.1752-4571.2007.00013.x PMC3352395 25567494

[35] Fritts HC (1976) Tree Rings and Climate. Academic Press, New York, NY, USA.

[36] Ma ZH, Peng CH, Zhu Q, Chen H, Yu GR, et al. (2012) Regional drought-induced reduction in the biomass carbon sink of Canada’s boreal forest. PNAS www.pnas.org/cgi/doi/10.1073/pnas.1111576109. 10.1073/pnas.1111576109 PMC3289349 22308340

[37] Thomson AM, Riddell CL, Parker WH (2009) Boreal forest provenance tests used to predict optimal growth and response to climate change: 2. *P. mariana.* . Can J For Res 39: 143–153.

[38] Eggers J, Lindner M, Zudin S, Zaehle S, Liski J (2008) Impact of changing wood demand, climate and land use on European forest resources and carbon stocks during the 21^st^ century. Global Change Biol 14: 2288–2303.

[39] Andalo C, Beaulieu J, Bousquet J (2005) The impact of climate change on growth of local white spruce populations in Quebec, Canada. For Ecol Manag 205: 169–182.

[40] Thomson AM, Parker WH (2008) Boreal forest provenance tests used to predict optimal growth and response to climate change: 1. *P. banksiana* . Can J For Res 38: 157–170.

[41] Peng CH, Ma ZH, Lei XD, Zhu Q, Chen H, et al.. (2011) A drought-induced pervasive increase in tree mortality across Canada’s boreal forest. Nature Climate Change doi:10.1038/nclimate1293.

[42] Reich PB, Oleksyn J (2008) Climate warming will reduce growth and survival of Scots pine except in the far north. Ecol Letters 11: 588–597. 10.1111/j.1461-0248.2008.01172.x 18363717

[43] Matyas C (1994) Modeling climate change effects with provenance test data. Tree Physiol 14: 797–804. 14967649 10.1093/treephys/14.7-8-9.797

[44] Kramer P, Kozlowski T (1979) Physiology of woody plants. Academic Press, San Diego, CA, USA.

[45] Kurz W, Dymond CC, Stinson G, Rampley GJ, Neilson ET, et al (2008) Mountain pine beetle and forest carbon feedback to climate change. Nature 452: 987–990. 18432244 10.1038/nature06777

[46] Gauthier S, Simon JP, Bergeron Y (1992) Genetic structure and variability in jack pine populations: effects of insularity. Can J For Res 22: 1958–1965.

[47] Petit RJ, Aguinagalde I, de Beaulieu JL, Bittkau C, Brewer S, et al (2003) Glacial refugia: hotspots but not melting pots of genetic diversity. Science 300: 1563–1565. 12791991 10.1126/science.1083264

[48] Huang JG, Bergeron Y, Zhai LH, Denneler B (2011) Variation in intra-annual radial growth (xylem formation) of *Picea mariana* (Pinaceae) along a latitudinal gradient in western Quebec, Canada. Amer J Botany 98: 792–800. 21613181 10.3732/ajb.1000074

[49] Zhai LH, Bergeron Y, Huang JG, Berninger F (2012) Variation in intra-annual wood formation, and foliage and shoot development of three major Canadian boreal tree species. Amer J Botany 99(5): 827–837. 22523348 10.3732/ajb.1100235

[50] Morin X, Lechowicz MJ, Augspurger C, O’Keefe J, Viner D, et al (2009) Leaf phenology in 22 North American tree species during the 21^st^ century. Global Change Biol 15: 961–975.

[51] Savolainen O, Pyhäjärvi T, Knürr T (2007) Gene flow and local adaptation in trees. Annu Rev Ecol Evol Syst 38: 595–619.

[52] Stadt KJ, Huston C, Coates KD, Feng Z, Dale MRT, et al (2007) Evaluation of competition and light estimation indices for predicting diameter growth in mature boreal mixed forests. Ann For Sci 64: 477–490.

[53] Huang JG, Bergeron Y, Denneler B, Berninger F, Tardif J (2007) Response of forest trees to increased atmospheric CO_2_ . Crit Rev Plant Sci 26: 265–283.

[54] Köchy M, Wilson SD (2001) Nitrogen deposition and forest expansion in the northern Great Plains. J Ecol 89: 807–817.

[55] Goldblum D, Rigg LS (2005) Tree growth response to climate change at the deciduous boreal forest ecotone, Ontario, Canada. Can J For Res 35: 2709–2718.

