# Supplementary figures and images for: Impact of Future Climate on Radial Growth of Four Major Boreal Tree Species in the Eastern Canadian Boreal Forest
Source: PLoS One. 2013 Feb 28;8(2):e56758. doi: 10.1371/journal.pone.0056758 (PMC3585260; doi:10.1371/journal.pone.0056758)

**Fig. S2**

**
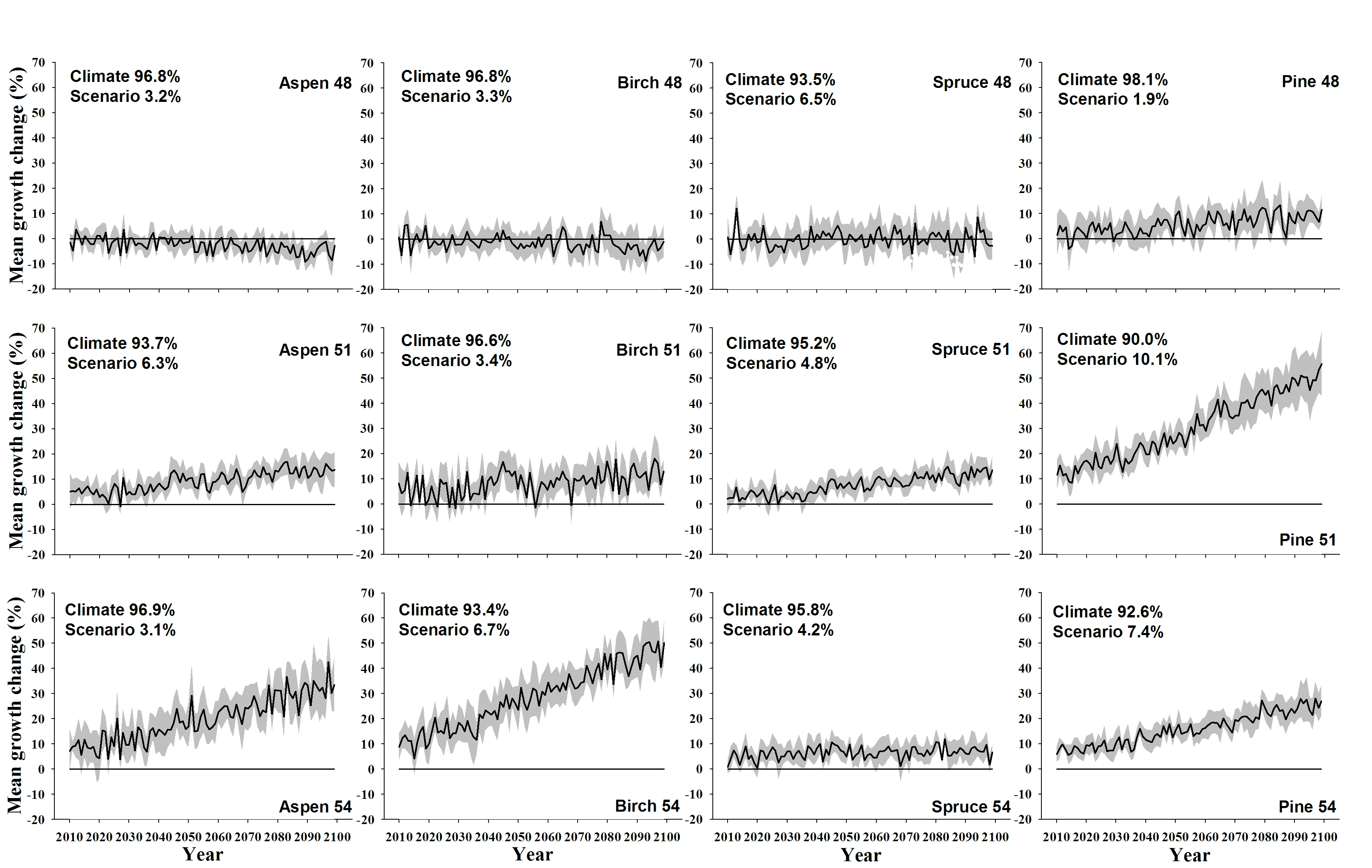
**


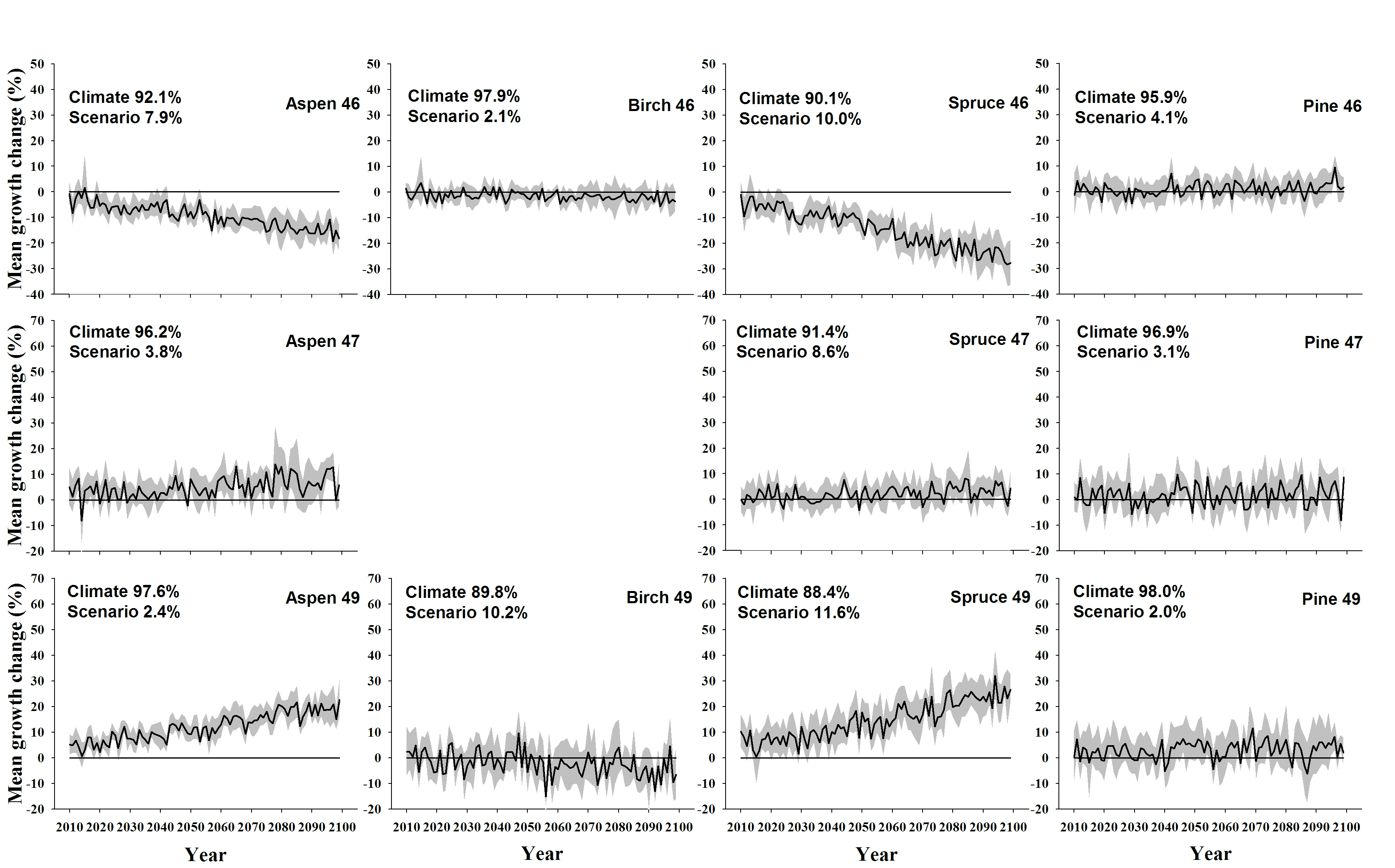


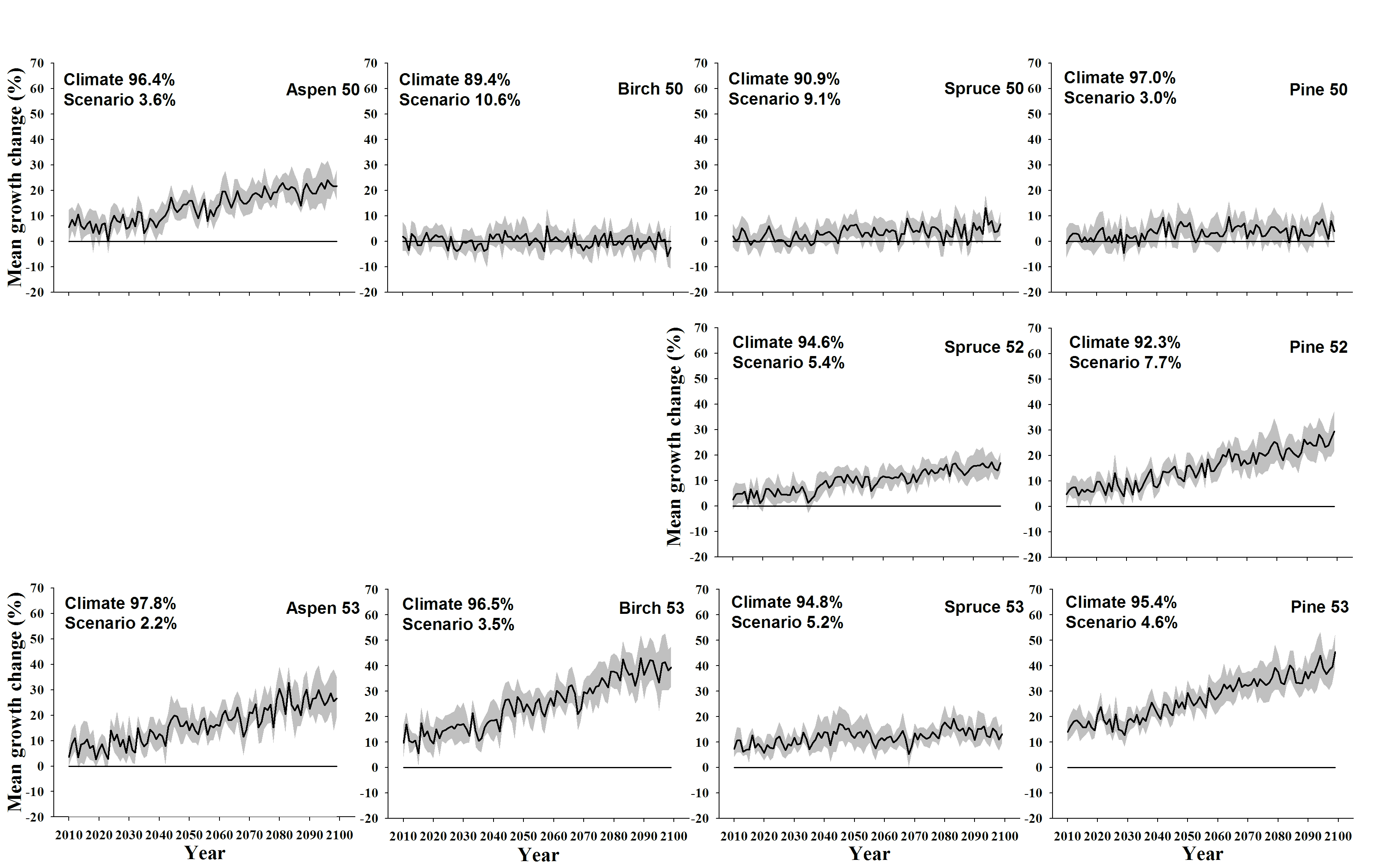

Supplement: Figure S2 — The predicted mean growth change of trembling aspen, paper birch, black spruce, and jack pine at 46–54°N under the F-model. The calibrated model for paper birch at 47°N was not established and thus the predicted mean growth change was not shown. (DOCX) [file pone.0056758.s002.docx]
