# Supplementary material for: Impact of Future Climate on Radial Growth of Four Major Boreal Tree Species in the Eastern Canadian Boreal Forest
Source: PLoS One. 2013 Feb 28;8(2):e56758. doi: 10.1371/journal.pone.0056758 (PMC3585260; doi:10.1371/journal.pone.0056758)
Supplement: Table S1 — General circulation models (GCM) including Canadian third-generation coupled global climate model (CGCM3), UK Hadley Centre HadCM3, Max Planck Institut für Meteorologie ECHAM4 and Canadian Regional Climate Model (CRCM3), their corresponding scenarios and the storylines (from the worst to the best) that describe the relationships between the forces driving greenhouse gas and aerosol emissions and their evolution during the 21st century applied in this study. (DOCX) [file pone.0056758.s003.docx]

**Table S1**.

| **GCMs** | **Scenarios** | **Acronyms** | **Storylines** |
| --- | --- | --- | --- |
| CGCM3 | A2 | CA2 | Intense forcing: a very heterogeneous world with continuously increasing global population and regionally oriented economic growth that is more fragmented and slower than in other storylines. |
|  | A1B | CA1B | Intense forcing: a future world of very rapid economic growth, global population that peaks in mid-century and declines thereafter, and rapid introduction of new and more efficient technologies. |
|  | B1 | CB1 | Intermediate forcing: a convergent world with the same global population as in the A1 storyline but with rapid changes in economic structures toward a service and information economy, with reductions in material intensity, and the introduction of clean and resource-efficient technologies. |
| HadCM3 | A2 | HA2 | See above |
|  | B2 | HB2 | Intermediate forcing: a world in which the emphasis is on local solutions to economic, social, and environmental sustainability, with continuously increasing population (lower than A2) and intermediate economic development. |
| ECHAM4 | A2 | EA2 | See above |
|  | B2 | EB2 | See above |
| CRCM3 | A2 | MA2 | See above |
