# Supplementary material for: Impact of Future Climate on Radial Growth of Four Major Boreal Tree Species in the Eastern Canadian Boreal Forest
Source: PLoS One. 2013 Feb 28;8(2):e56758. doi: 10.1371/journal.pone.0056758 (PMC3585260; doi:10.1371/journal.pone.0056758)
Supplement: Table S2 — Statistics of the model calibration and verification for trembling aspen, paper birch, black spruce and jack pine along the gradient. Significance level is at p<0.5. Note: r: correlation coefficient; R2: explained variance; adjR2: square of the multiple correlation coefficients following adjustment for loss of degrees of freedom; SE: standard error of the predictions; RE: reduction of error statistic, which is a measure of shared variance between the actual and modelled series, but is usually lower than the calibration R2. A positive value signifies that the regression model has some skill [24]. PM: product means test [35]; ST: sign test [35]. The italic texts indicate insignificant values. (DOCX) [file pone.0056758.s004.docx]

**Table S2**

| **Latitude**  **(ºN)** | **Calibration_Aspen** |  |  |  | **Verification_Aspen** |  |  |  |  |  | **Calibration_Birch** |  |  |  | **Verification_Birch** |  |  |  |  |
| --- | --- | --- | --- | --- | --- | --- | --- | --- | --- | --- | --- | --- | --- | --- | --- | --- | --- | --- | --- |
|  | Period | R^2^ | adjR^2^ | SE | Period | r | RE | PM | ST (+/-) |  | Period | R^2^ | adjR^2^ | SE | Period | r | RE | PM | ST (+/-) |
| 46 | 1912-1965 | 0.22 | 0.14 | .09 | 1966-2003 | 0.29 | *0.04* | 1.70 | 21/17 |  | 1905-1965 | 0.27 | 0.22 | .07 | 1966-2003 | 0.26 | *0.03* | 1.89 | 23/15 |
|  | 1966-2003 | 0.21 | 0.13 | .08 | 1912-1965 | 0.27 | *0.04* | 2.03 | 28/26 |  | 1966-2003 | 0.20 | 0.15 | .09 | 1905-1965 | 0.49 | 0.21 | 4.09 | 36/25 |
|  | 1912-2003 | 0.21 | 0.16 | .08 |  |  |  |  |  |  | 1905-2003 | 0.18 | 0.15 | .08 |  |  |  |  |  |
|  |  |  |  |  |  |  |  |  |  |  |  |  |  |  |  |  |  |  |  |
| 47 | 1902-1965 | 0.32 | 0.25 | .06 | 1966-2003 | 0.36 | *0.05* | 4.63 | 25/13 |  | 1902-1965 | NA | NA | NA | 1966-2003 | NA | NA | NA | NA |
|  | 1966-2003 | 0.31 | 0.25 | .08 | 1902-1965 | 0.15 | 0.19 | 1.29 | 35/29 |  | 1966-2003 | NA | NA | NA | 1902-1965 | NA | NA | NA | NA |
|  | 1902-2003 | 0.27 | 0.23 | .09 |  |  |  |  |  |  | 1902-2003 | NA | NA | NA |  | NA | NA | NA | NA |
|  |  |  |  |  |  |  |  |  |  |  |  |  |  |  |  |  |  |  |  |
| 48 | 1910-1965 | 0.20 | 0.16 | .08 | 1966-2003 | 0.46 | 0.17 | 5.72 | 27/11 |  | 1907-1965 | 0.24 | 0.21 | .08 | 1966-2003 | 0.22 | *0.02* | 2.05 | 20/18 |
|  | 1966-2003 | 0.40 | 0.36 | .07 | 1910-1965 | 0.29 | 0.05 | 2.82 | 35/21 |  | 1966-2003 | 0.16 | 0.13 | .06 | 1907-1965 | 0.46 | 0.21 | 6.99 | 41/18 |
|  | 1910-2003 | 0.33 | 0.29 | .08 |  |  |  |  |  |  | 1907-2003 | 0.20 | 0.17 | .07 |  |  |  |  |  |
|  |  |  |  |  |  |  |  |  |  |  |  |  |  |  |  |  |  |  |  |
| 49 | 1915-1965 | 0.29 | 0.23 | .08 | 1966-2003 | 0.26 | 0.25 | 7.36 | 30/8 |  | 1912-1965 | 0.17 | 0.14 | .07 | 1966-2003 | 0.53 | 0.28 | 6.32 | 28/10 |
|  | 1966-2003 | 0.14 | 0.12 | .06 | 1915-1965 | 0.46 | 0.29 | 10.33 | 38/13 |  | 1966-2003 | 0.29 | 0.21 | .06 | 1912-1965 | 0.40 | 0.16 | 5.12 | 33/21 |
|  | 1915-2003 | 0.26 | 0.22 | .08 |  |  |  |  |  |  | 1912-2003 | 0.21 | 0.18 | .05 |  |  |  |  |  |
|  |  |  |  |  |  |  |  |  |  |  |  |  |  |  |  |  |  |  |  |
| 50 | 1902-1965 | 0.15 | 0.11 | .09 | 1966-2003 | 0.60 | 0.29 | 7.15 | 26/12 |  | 1928-1965 | 0.36 | 0.30 | .06 | 1966-2003 | 0.59 | 0.24 | 8.40 | 32/6 |
|  | 1966-2003 | 0.42 | 0.37 | .07 | 1902-1965 | 0.29 | 0.13 | 2.61 | 41/23 |  | 1966-2003 | 0.31 | 0.27 | .06 | 1928-1965 | 0.50 | 0.20 | 4.83 | 20/18 |
|  | 1902-2003 | 0.23 | 0.20 | .09 |  |  |  |  |  |  | 1928-2003 | 0.33 | 0.31 | .07 |  |  |  |  |  |
|  |  |  |  |  |  |  |  |  |  |  |  |  |  |  |  |  |  |  |  |
| 51 | 1929-1965 | 0.18 | 0.15 | .06 | 1966-2003 | 0.62 | 0.36 | 8.29 | 26/12 |  | 1909-1965 | 0.47 | 0.39 | .07 | 1966-2003 | 0.31 | 0.11 | 3.01 | 22/16 |
|  | 1966-2003 | 0.36 | 0.30 | .07 | 1929-1965 | 0.32 | *0.01* | 3.01 | 24/13 |  | 1966-2003 | 0.25 | 0.20 | .05 | 1909-1965 | 0.38 | *0.03* | 2.51 | 31/26 |
|  | 1929-2003 | 0.29 | 0.25 | .07 |  |  |  |  |  |  | 1909-2003 | 0.32 | 0.26 | .06 |  |  |  |  |  |
|  |  |  |  |  |  |  |  |  |  |  |  |  |  |  |  |  |  |  |  |
| 53 | 1926-1965 | 0.52 | 0.46 | .07 | 1966-2003 | 0.29 | *0.05* | 3.18 | 21/17 |  | 1933-1965 | 0.47 | 0.38 | .04 | 1966-2003 | 0.55 | 0.26 | 7.26 | 23/15 |
|  | 1966-2003 | 0.32 | 0.24 | .08 | 1926-1965 | 0.51 | 0.32 | 8.45 | 26/14 |  | 1966-2003 | 0.50 | 0.43 | .05 | 1933-1965 | 0.58 | 0.50 | 4.81 | 22/11 |
|  | 1926-2003 | 0.38 | 0.32 | .08 |  |  |  |  |  |  | 1933-2003 | 0.40 | 0.35 | .05 |  |  |  |  |  |
|  |  |  |  |  |  |  |  |  |  |  |  |  |  |  |  |  |  |  |  |
| 54 | 1927-1965 | 0.37 | 0.30 | .07 | 1966-2003 | 0.46 | *0.03* | 3.48 | 24/14 |  | 1931-1965 | 0.21 | 0.18 | .05 | 1966-2003 | 0.59 | 0.33 | 9.22 | 28/10 |
|  | 1966-2003 | 0.38 | 0.35 | .08 | 1927-1965 | 0.34 | 0.12 | 4.43 | 25/14 |  | 1966-2003 | 0.40 | 0.37 | .05 | 1931-1965 | 0.30 | *0.03* | 2.47 | 21/14 |
|  | 1927-2003 | 0.34 | 0.30 | .08 |  |  |  |  |  |  | 1927-2003 | 0.24 | 0.22 | .04 |  |  |  |  |  |

| **Latitude**  **(ºN)** | **Calibration_Spruce** |  |  |  | **Verification_Spruce** |  |  |  |  |  | **Calibration_Pine** |  |  |  | **Verification_Pine** |  |  |  |  |
| --- | --- | --- | --- | --- | --- | --- | --- | --- | --- | --- | --- | --- | --- | --- | --- | --- | --- | --- | --- |
|  | Period | R^2^ | adjR^2^ | SE | Period | r | RE | PM | ST (+/-) |  | Period | R^2^ | adjR^2^ | SE | Period | r | RE | PM | ST (+/-) |
| 46 | 1946-1965 | 0.22 | 0.18 | .05 | 1966-2003 | 0.32 | 0.07 | 2.20 | 24/14 |  | 1926-1965 | 0.36 | 0.27 | .04 | 1966-2003 | 0.48 | 0.21 | 7.65 | 27/11 |
|  | 1966-2003 | 0.26 | 0.20 | .06 | 1946-1965 | 0.50 | 0.26 | 7.00 | 13/7 |  | 1966-2003 | 0.32 | 0.25 | .04 | 1926-1965 | 0.41 | 0.65 | 3.00 | 28/12 |
|  | 1946-2003 | 0.26 | 0.22 | .06 |  |  |  |  |  |  | 1926-2003 | 0.30 | 0.24 | .03 |  |  |  |  |  |
|  |  |  |  |  |  |  |  |  |  |  |  |  |  |  |  |  |  |  |  |
| 47 | 1902-1965 | 0.31 | 0.25 | .07 | 1966-2003 | 0.37 | *0.01* | 3.15 | 26/12 |  | 1902-1965 | 0.33 | 0.30 | .04 | 1966-2003 | 0.42 | 0.11 | 5.93 | 25/13 |
|  | 1966-2003 | 0.20 | 0.15 | .08 | 1902-1965 | 0.41 | 0.12 | 3.00 | 45/19 |  | 1966-2003 | 0.31 | 0.23 | .05 | 1902-1965 | 0.43 | 0.16 | 4.13 | 42/22 |
|  | 1902-2003 | 0.26 | 0.22 | .03 |  |  |  |  |  |  | 1902-2003 | 0.38 | 0.34 | .03 |  |  |  |  |  |
|  |  |  |  |  |  |  |  |  |  |  |  |  |  |  |  |  |  |  |  |
| 48 | 1902-1965 | 0.30 | 0.23 | .06 | 1966-2003 | 0.48 | 0.17 | 5.34 | 25/13 |  | 1920-1965 | 0.40 | 0.37 | .03 | 1966-2003 | 0.55 | 0.22 | 5.62 | 26/12 |
|  | 1966-2003 | 0.24 | 0.17 | .05 | 1902-1965 | 0.43 | 0.16 | 3.25 | 39/25 |  | 1966-2003 | 0.57 | 0.52 | .03 | 1920-1965 | 0.55 | 0.26 | 10.43 | 30/16 |
|  | 1902-2003 | 0.26 | 0.21 | .05 |  |  |  |  |  |  | 1920-2003 | 0.50 | 0.46 | .02 |  |  |  |  |  |
|  |  |  |  |  |  |  |  |  |  |  |  |  |  |  |  |  |  |  |  |
| 49 | 1919-1965 | 0.25 | 0.17 | .04 | 1966-2003 | 0.41 | 0.16 | 4.23 | 24/14 |  | 1913-1965 | 0.45 | 0.39 | .03 | 1966-2003 | 0.59 | 0.33 | 9.08 | 25/13 |
|  | 1966-2003 | 0.44 | 0.36 | .03 | 1919-1965 | 0.35 | 0.14 | 2.68 | 28/19 |  | 1966-2003 | 0.38 | 0.33 | .03 | 1913-1965 | 0.57 | 0.20 | 5.94 | 38/15 |
|  | 1919-2003 | 0.33 | 0.26 | .03 |  |  |  |  |  |  | 1913-2003 | 0.40 | 0.36 | .02 |  |  |  |  |  |
|  |  |  |  |  |  |  |  |  |  |  |  |  |  |  |  |  |  |  |  |
| 50 | 1902-1965 | 0.22 | 0.17 | .05 | 1966-2003 | 0.34 | 0.11 | 3.24 | 23/15 |  | 1917-1965 | 0.30 | 0.26 | .01 | 1966-2003 | 0.53 | 0.26 | 6.97 | 23/15 |
|  | 1966-2003 | 0.23 | 0.19 | .05 | 1902-1965 | 0.42 | 0.18 | 5.51 | 42/22 |  | 1966-2003 | 0.51 | 0.47 | .02 | 1917-1965 | 0.38 | 0.08 | 3.58 | 30/19 |
|  | 1902-2003 | 0.23 | 0.19 | .03 |  |  |  |  |  |  | 1917-2003 | 0.38 | 0.35 | .01 |  |  |  |  |  |
|  |  |  |  |  |  |  |  |  |  |  |  |  |  |  |  |  |  |  |  |
| 51 | 1902-1965 | 0.23 | 0.19 | .06 | 1966-2003 | 0.42 | 0.15 | 3.48 | 24/14 |  | 1938-1965 | 0.48 | 0.41 | .02 | 1966-2003 | 0.37 | 0.09 | 3.68 | 24/14 |
|  | 1966-2003 | 0.26 | 0.20 | .05 | 1902-1965 | 0.44 | *0.02* | 4.54 | 37/27 |  | 1966-2003 | 0.24 | 0.20 | .02 | 1938-1965 | 0.57 | 0.32 | 8.55 | 22/6 |
|  | 1902-2003 | 0.27 | 0.23 | .05 |  |  |  |  |  |  | 1938-2003 | 0.32 | 0.27 | .01 |  |  |  |  |  |
|  |  |  |  |  |  |  |  |  |  |  |  |  |  |  |  |  |  |  |  |
| 52 | 1902-1965 | 0.22 | 0.18 | .04 | 1966-2003 | 0.43 | 0.14 | 4.13 | 29/9 |  | 1938-1965 | 0.30 | 0.28 | .03 | 1966-2003 | 0.42 | 0.09 | 3.06 | 23/15 |
|  | 1966-2003 | 0.19 | 0.15 | .05 | 1902-1965 | 0.34 | *0.02* | 3.06 | 37/27 |  | 1966-2003 | 0.40 | 0.33 | .02 | 1938-1965 | 0.54 | *0.06* | 3.19 | 18/10 |
|  | 1902-2003 | 0.24 | 0.21 | .03 |  |  |  |  |  |  | 1938-2003 | 0.34 | 0.30 | .02 |  |  |  |  |  |
|  |  |  |  |  |  |  |  |  |  |  |  |  |  |  |  |  |  |  |  |
| 53 | 1911-1965 | 0.26 | 0.21 | .04 | 1966-2003 | 0.52 | 0.20 | 4.75 | 25/13 |  | 1902-1965 | 0.47 | 0.44 | .03 | 1966-2003 | 0.55 | 0.23 | 5.37 | 28/10 |
|  | 1966-2003 | 0.29 | 0.23 | .04 | 1911-1965 | 0.48 | 0.06 | 4.56 | 31/24 |  | 1966-2003 | 0.48 | 0.46 | .04 | 1902-1965 | 0.26 | 0.29 | 1.99 | 38/26 |
|  | 1911-2003 | 0.25 | 0.22 | .03 |  |  |  |  |  |  | 1902-2003 | 0.42 | 0.39 | .03 |  |  |  |  |  |
|  |  |  |  |  |  |  |  |  |  |  |  |  |  |  |  |  |  |  |  |
| 54 | 1902-1965 | 0.24 | 0.17 | .04 | 1966-2003 | 0.46 | 0.20 | 4.22 | 27/11 |  | 1918-1965 | 0.24 | 0.16 | .04 | 1966-2003 | 0.49 | 0.19 | 7.04 | 24/14 |
|  | 1966-2003 | 0.27 | 0.16 | .05 | 1902-1965 | 0.35 | 0.09 | 3.78 | 44/20 |  | 1966-2003 | 0.39 | 0.35 | .03 | 1918-1965 | 0.32 | 0.25 | 3.19 | 18/10 |
|  | 1902-2003 | 0.23 | 0.19 | .03 |  |  |  |  |  |  | 1918-2003 | 0.24 | 0.20 | .02 |  |  |  |  |  |
