# Supplementary material for: Impact of Future Climate on Radial Growth of Four Major Boreal Tree Species in the Eastern Canadian Boreal Forest
Source: PLoS One. 2013 Feb 28;8(2):e56758. doi: 10.1371/journal.pone.0056758 (PMC3585260; doi:10.1371/journal.pone.0056758)
Supplement: Table S3 — The calibrated full-period climate-growth (Tree-Ring Index, TRI) models for trembling aspen, paper birch, black spruce, and jack pine along the latitudinal gradient from 46°N to 54°N. Note: The chronology full period and adj R2 of each model were listed in Table S2. Monthly climate variables were abbreviations in the model, for example climate variables in May, p5p and p5 indicates precipitation in the previous and current May, respectively; tmax5p and tmax5 indicates maximum temperature in the previous and current May, respectively; tmin5p and tmin5 indicates minimum temperature in the previous and current May, respectively; dc5p and dc5 indicates drought code in the previous and current May. (DOCX) [file pone.0056758.s005.docx]

**Table S3**

| **Latitude (ºN) / Species** | **Calibrated full-period climate-growth models along the latitudinal gradient** |
| --- | --- |
|  |  |
| 46 / Aspen | TRI=1.48482+0.00092552*(p5p+p6p+p7p+p8p+p9p)-0.00040307* (p3+p4+p5+p6+p7+p8+p9)-0.03997*[(tmax3+tmax4+tmax5+tmax6)/4]-0.04082*[(tmin8p+tmin9p+tmin10p+tmin11p)/4]+0.03366*[(tmin2+tmin3+tmin4+tmin5+tmin6)/5]+0.00078905*[(dc7p+dc8p+dc9p+dc10p)/4] |
|  |  |
| 47 / Aspen | TRI =0.84163+0.00064779*(p9p+p10p+p11p)-0.00090518*(p4+p5+p6+p7+p8+p9)+0.03218*[(tmax3+tmax4+tmax5)/3]+0.04228*[(tmax7+tmax8)/2]-0.04802*[(tmin6+tmin7+tmin8+tmin9)/4]-0.00193*[(dc6+dc7+dc8)/3] |
|  |  |
| 48 / Aspen | TRI =1.20669-0.00050084*(p11p+p12p+p1+p2)+0.05291*[(tmax12p+tmax1+tmax2+tmax3+tmax4+tmax5)/6]-0.02077*[(tmax9p+tmax10p+tmax11p)/3]-0.0147*tmin5p-0.01294*[(tmin12p+tmin1)/2]-0.00059883*[(dc5+dc6+dc7+dc8)/4] |
|  |  |
| 49 / Aspen | TRI =1.19092-0.02183*[(tmax1+tmax2)/2]+0.03537*[(tmin6p+tmin7p+tmin8p+tmin9p+tmin10p+tmin11p+tmin12p)/7]+0.03398*[(tmin2+tmin3+tmin4+tmin5+tmin6)/5]-0.00109*[(dc5+dc6+dc7+dc8)/4] |
|  |  |
| 50 / Aspen | TRI =0.78183+0.00125*(p5p+p6p)-0.00111*p8+0.00072492*(p3+p4+p5+p6+p7)+0.02968*[(tmin3+tmin4+tmin5+tmin6+tmin7)/5] |
|  |  |
| 51 / Aspen | TRI =0.59569+0.00106*(p5p+p6p+p7p)+0.0006362*(p4+p5+p6+p7+p8)-0.03022*[(tmax6p+tmax7p+tmax8p+tmax9p+tmax10p+tmax11p)/6]+0.0513*[(tmin6+tmin7+tmin8+tmin9)/4] |
|  |  |
| 53 / Aspen | TRI =0.53665+0.00153*(p3+p4+p5+p6+p7+p8)-0.06643*[(tmax12p+tmax1+tmax2)/3]+0.05133*[(tmin12p+tmin1+tmin2)/3]+0.05593*[(tmin6+tmin7+tmin8)/3]-0.00118*[(dc5p+dc6p+dc7p+dc8p+dc9p+dc10p)/6]+0.0011*[(dc5+dc6+dc7+dc8+dc9)/5] |
|  |  |
| 54 / Aspen | TRI =0.76525+0.00276*p5p-0.00134*p9+0.01517*tmax3+0.06864*[(tmin7+ tmin8)/2]-0.00075441*[(dc8p+dc9p+dc10p)/3] |

| **Latitude (ºN) / Species** | **Calibrated full-period climate-growth models along the latitudinal gradient** |
| --- | --- |
|  |  |
| 46 / Birch | TRI =1.49196+0.00097013*(p7p+p8p+p9p)-0.06971*[(tmax2+tmax3+tmax4+tmax5+tmax6)/5]+0.05194*[(tmin2+tmin3+tmin4+tmin5+tmin6)/5]+0.00058924*dc10p |
|  |  |
| 48 / Birch | TRI =1.39767-0.00127*(p8+p9)-0.00149*[(dc6+dc7+dc8+dc9)/4]+0.00213*dc5 |
|  |  |
| 49 / Birch | TRI =1.5191-0.07925*[(tmax1+tmax2+tmax3+tmax4+tmax5+tmax6)/6]+0.1167*[(tmin3+tmin4+tmin5+tmin6+tmin7+tmin8)/6]-0.0467*tmin9-0.00122*[(dc5+dc6+dc7+dc8)/4] |
|  |  |
| 50 / Birch | TRI =2.02279+0.02234*tmax5p+0.02508*[(tmax12p+tmax1+tmax2)/3]-0.05002*[(tmax6p+tmax7p+tmax8p)/3] |
|  |  |
| 51 / Birch | TRI =1.00859+0.00127*(p5+p6+p7+p8)-0.00165*p9+0.06151*[(tmax7+tmax8+tmax9)/3]-0.05855*[(tmax3+tmax4+tmax5+tmax6)/4]-0.03045*[(tmax6p+tmax7p+tmax8p)/3]+0.07503*[(tmin3+tmin4+tmin5+tmin6+tmin7)/5]-0.04072*tmin9 |
|  |  |
| 53 / Birch | TRI =0.2017+0.00099563*(p4+p5+p6+p7+p8)+0.01445*tmax5p+0.04589*[(tmax6+tmax7+tmax8+tmax9)/4] +0.0272*[(tmin9p+tmin10p+tmin11p+tmin12p)/4]-0.00196*[(dc5p+dc6p+dc7p)/3] |
|  |  |
| 54 / Birch | TRI =1.39771+0.07637*[(tmax9p+tmax10p+tmax11p+tmax12p+tmax1+tmax2+tmax3)/7]-0.00074111*[(dc8p+dc9p+dc10p)/3] |

| **Latitude (ºN) / Species** | **Calibrated full-period climate-growth models along the latitudinal gradient** |
| --- | --- |
|  |  |
| 46 / Spruce | TRI =2.16894+0.05301*[(tmax11p+tmax12p)/2]-0.05155*[(tmax6+tmax7+tmax8+tmax9)/4]-0.05799*[(tmin9p+tmin10p+tmin11p+tmin12p)/4] |
|  |  |
| 47 / Spruce | TRI =1.57534-0.00092835*(p5+p6)+0.02562*[(tmax8+tmax9)/2]+0.02739*[(tmin2+tmin3+tmin4)/3]-0.04264*[(tmin6+tmin7+tmin8)/3]-0.00105*[(dc6+dc7+dc8)/3] |
|  |  |
| 48 / Spruce | TRI =1.86219+0.00095323*(p5p+p6p+p7p)-0.03547*[(tmax7+tmax8+tmax9)/3]+0.02419*[(tmin12p+tmin1+tmin2+tmin3+tmin4)/5]-0.0012*[(dc6+dc7+dc8)/3]+ 0.00224*dc5+0.00057414*dc9 |
|  |  |
| 49 / Spruce | TRI =1.90837+0.00071661*(p5p+p6p+p7p)-0.00050706*(p4+p5+p6+p7+p8)-0.00108*(p8p+p9p+p10p)+0.02955*[(tmax9p+tmax10p+tmax11p+tmax12p)/4]-0.04644*[(tmax5+tmax6+tmax7+tmax8)/4]+0.07141*[(tmin4+tmin5+tmin6+tmin7+tmin8)/5] +0.00089305*[(dc7p+dc8p+dc9p)/3]-0.00126*dc10p |
|  |  |
| 50 / Spruce | TRI =1.50701+0.00094755*(p5p+p6p)+0.03345*[(tmax12p+tmax1+tmax2+tmax3+tmax4+tmax5)/6]-0.03636*[(tmax6+tmax7+tmax8+tmax9)/4]+0.00045703*[(dc7p+dc8p)/2]+0.00068851*[(dc5+dc6+dc7+dc8)/4] |
| 51 / Spruce | TRI=0.74844+0.00059553*(p5p+p6p+p7p+p8p+p9p+p10p)+0.05205*[(tmax9p+tmax10p+tmax11p+tmax12p+tmax1+tmax2+tmax3+tmax4+tmax5)/9]-0.0354*[(tmin5p+tmin6p+tmin7p+tmin8p+tmin9p+tmin10p+tmin11p+tmin12p)/8]-0.0007636*[(dc5p+dc6p+dc7p+dc8p)/4]+0.00042744*[(dc9p+dc10p)/2] |
|  |  |
| 52 / Spruce | TRI =1.01148+0.00056153*(p5+p6+p7+p8)+0.02607*[(tmax1+tmax2+tmax3+tmax4+tmax5)/5]-0.00105*[(dc5p+dc6p+dc7p+dc8p+dc9p)/5]+0.00037843*dc10p |
|  |  |
| 53 / Spruce | TRI =0.55886+0.00101*(p6+p7+p8)+0.01962*[(tmax6+tmax7+tmax8+tmax9)/4]-0.00098141*[(dc5p+dc6p+dc7p+dc8p+dc9p)/5] |
|  |  |
| 54 / Spruce | TRI =1.09988-0.00063977*(p11p+p12p+p1)+0.00088221*(p6+p7+p8)-0.00095704*p9+0.00844*[(tmin3+tmin4)/2]-0.0006434*[(dc5p+dc6p+dc7p+dc8p+dc9p+dc10p)/6]+0.00067757*[(dc6+dc7)/2] |

| **Latitude (ºN) / Species** | **Calibrated full-period climate-growth models along the latitudinal gradient** |
| --- | --- |
|  |  |
| 46 / Pine | TRI =1.50634-0.06341*[(tmax9p+tmax10p+tmax11p+tmax12p+tmax1)/5]+0.03637*[(tmax2+tmax3+tmax4+tmax5+tmax6+tmax7)/6]-0.03229*[(tmin6p+tmin7p+tmin8p+tmin9p)/4]+0.04769*[(tmin10p+tmin11p+tmin12p+tmin1)/4]-0.00072391*[(dc5p+dc6p+dc7p)/3]+0.00072059*[(dc8p+dc9p+dc10p)/3] |
|  |  |
| 47 / Pine | TRI =1.35416+0.04425*[(tmax2+tmax3+tmax4+tmax5+tmax6)/5]-0.05398*[(tmin7p+tmin8p+tmin9p)/3]+0.02386*[(tmin10p+tmin11p)/2]+0.00075418*[(dc7p+dc8p+dc9p)/3]-0.00044001*dc10p-0.00156*[(dc5+dc6+dc7+dc8)/4] |
|  |  |
| 48 / Pine | TRI =1.2853-0.00070978*(p4+p5+p6+p7+p8)+0.06574*[(tmax3+tmax4+tmax5+tmax6)/4]-0.05006*[(tmin7p+tmin8p+tmin9p)/3]+0.02151*[(tmin10p+tmin11p)/2]-0.00154*[(dc5+dc6+dc7+dc8)/4] |
|  |  |
| 49 / Pine | TRI =0.5291+0.0007898*(p5p+p6p+p7p+p8p+p9p)+0.02997*[(tmax10p+tmax11p)/2]+0.04688*[(tmax3+tmax4+tmax5+tmax6)/4]-0.0726*[(tmin7p+tmin8p+tmin9p)/3]+0.00082412*dc10p |
|  |  |
| 50 / Pine | TRI =1.5187-0.00109*(p10p+p11p+p12p)+0.02977* [(tmax11p+tmax12p+tmax1+tmax2+tmax3)/5]-0.04198*[(tmin8p+tmin9p+tmin10p)/3]+0.0273*[(tmin4+tmin5+tmin6+tmin7+tmin8)/5] |
|  |  |
| 51 / Pine | TRI =0.09386+0.03502*[(tmax8p+tmax9p+tmax10p)/3]+0.02065*tmax9+0.05463*[(tmin4+tmin5+tmin6+tmin7+tmin8)/5]+0.00092871*[(dc5+dc6+dc7+dc8)/4]-0.00060938*dc9 |
|  |  |
| 52 / Pine | TRI =0.33041+0.00261*p3+0.05238*[(tmax8p+tmax9p+tmax10p)/3]-0.05803*[(tmin8p+tmin9p+tmin10p)/3]+0.05373*[(tmin4+tmin5+tmin6+tmin7+tmin8)/5] |
|  |  |
| 53 / Pine | TRI =0.85127+0.00158*(p1+p2+p3)+0.0486*[(tmax4+tmax5+tmax6+tmax7)/4]-0.06068*[(tmax8+tmax9)/2]+0.06534*[(tmin8+tmin9)/2] |
|  |  |
| 54 / Pine | TRI =1.13453+0.01976*[(tmax5p+tmax6p)/2]+0.02507*[(tmax4+tmax5+tmax6)/3]-0.0553*[(tmax7+tmax8+tmax9)/3]+0.07702*[(tmin7+tmin8+tmin9)/3]-0.00108*[(dc5p+dc6p+dc7p+dc8p)/4] |
