# Supplementary material for: Impact of Future Climate on Radial Growth of Four Major Boreal Tree Species in the Eastern Canadian Boreal Forest
Source: PLoS One. 2013 Feb 28;8(2):e56758. doi: 10.1371/journal.pone.0056758 (PMC3585260; doi:10.1371/journal.pone.0056758)
Supplement: Table S4 — The mean climate values (±SD) during the reference period of 1961–1990 over the latitudinal gradient 46–54°N in eastern Canada. Note: Max T and Min T: Mean maximum, and minimum temperature during 1961–90; Precipitation: mean total annual precipitation during 1961–90; Drought code is mean drought code during 1961–90. (DOCX) [file pone.0056758.s006.docx]

**Table S4**.

| **Latitude (ºN)** | **Max T (°C)** | **Min T (°C)** | **Precipitation (mm)** | **Drought code (unit)** |
| --- | --- | --- | --- | --- |
| 46 | 8.4 (± 11.0) | -2.0 (± 10.8) | 936.6 (± 29.7) | 281.3 (± 188.7) |
| 47 | 7.1 (± 11.2) | -3.0 (± 11.0) | 830.5 (± 29.0) | 296.5 (± 195.7) |
| 48 | 6.3 (± 11.4) | -3.7 (± 11.3) | 818.7 (± 29.0) | 294.2(± 194.3) |
| 49 | 5.1 (± 11.5) | -4.7 (± 11.6) | 821.1 (± 30.4) | 277.7 (± 180.9) |
| 50 | 4.0 (± 11.6) | -5.6 (± 11.8) | 791.3 (± 32.0) | 245.8 (± 160.4) |
| 51 | 1.4 (± 11.0) | -7.1 (± 11.4) | 765.6 (± 30.1) | 228.0 (± 151.0) |
| 52 | -0.8 (± 10.5) | -8.2 (± 11.2) | 725.6 (± 29.4) | 197.3 (± 134.0) |
| 53 | -1.2 (± 10.4) | -8.5 (± 11.2) | 722.2 (± 29.7) | 190.1 (± 130.0) |
| 54 | -3.0 (± 10.0) | -9.3 (± 11.0) | 673.1 (± 29.8) | 150.7 (± 107.1) |
