# Supplementary material for: Impact of Future Climate on Radial Growth of Four Major Boreal Tree Species in the Eastern Canadian Boreal Forest
Source: PLoS One. 2013 Feb 28;8(2):e56758. doi: 10.1371/journal.pone.0056758 (PMC3585260; doi:10.1371/journal.pone.0056758)
Supplement: Table S5 — Mean annual ring width [RW (±SD) mm] growth of the four species from 1961 to 1990 over the latitudinal gradient 46–54°N in eastern Canada. Note: NA indicates that stands were not found at 52°N. (DOCX) [file pone.0056758.s007.docx]

**Table S5**.

| **Latitude** | **RW_Aspen** | **RW_Birch** | **RW_Spruce** | **RW_Pine** |
| --- | --- | --- | --- | --- |
| 46 | 2.131 (± 0.625) | 0.971 (± 0.401) | 1.381 (± 0.523) | 1.428 (± 0.401) |
| 47 | 2.172 (± 0.842) | 0.924 (± 0.538) | 0.638 (± 0.259) | 0.741 (± 0.244) |
| 48 | 2.789 (± 0.791) | 1.371 (± 0.532) | 0.740 (± 0.289) | 1.064 (± 0.306) |
| 49 | 1.648 (± 0.534) | 0.593 (± 0.276) | 0.705 (± 0.221) | 0.580 (± 0.243) |
| 50 | 1.118 (± 0.387) | 1.398 (± 0.469) | 0.788 (± 0.246) | 0.605 (± 0.189) |
| 51 | 1.495 (± 0.579) | 0.475 (± 0.328) | 0.387 (± 0.112) | 1.056 (± 0.507) |
| 52 | NA | NA | 0.796 (± 0.239) | 1.001 (± 0.431) |
| 53 | 1.376 (± 0.632) | 1.023 (± 0.423) | 0.941 (± 0.301) | 0.300 (± 0.146) |
| 54 | 1.587 (± 0.652) | 0.976 (± 0.664) | 0.569 (± 0.158) | 0.820 (± 0.513) |
